# Development of a multifunctional bioreactor to evaluate the promotion effects of cyclic stretching and electrical stimulation on muscle differentiation

**DOI:** 10.1002/btm2.10633

**Published:** 2023-12-07

**Authors:** Wei‐Wen Hu, Yen‐Chi Chen, Chia‐Wen Tsao, Shen‐Liang Chen, Chung‐Yuh Tzeng

**Affiliations:** ^1^ Department of Chemical and Materials Engineering National Central University Taoyuan Taiwan; ^2^ Department of Mechanical Engineering National Central University Taoyuan Taiwan; ^3^ Department of Life Sciences National Central University Taoyuan Taiwan; ^4^ Department of Orthopedics Taichung Veterans General Hospital Taichung Taiwan; ^5^ Department of Rehabilitation Jen‐Teh Junior College of Medicine, Nursing and Management Miaoli Taiwan; ^6^ Department of Medicinal Botanicals and Foods on Health Applications Da‐Yeh University Changhua Taiwan; ^7^ Institute of Biomedical Sciences National Chung Hsing University Taichung Taiwan

**Keywords:** bioreactor, cyclic stretching, electrical stimulation, myoblasts, myogenic differentiation, polydimethylsiloxane, polypyrrole

## Abstract

A multifunctional bioreactor was fabricated in this study to investigate the facilitation efficiency of electrical and mechanical stimulations on myogenic differentiation. This bioreactor consisted of a highly stretchable conductive membrane prepared by depositing polypyrrole (PPy) on a flexible polydimethylsiloxane (PDMS) film. The tensile deformation of the PPy/PDMS membrane can be tuned by adjusting the channel depth. In addition, PPy/PDMS maintained its electrical conductivity under continuous cyclic stretching in the strain range of 6.5%–13% for 24 h. This device can be used to individually or simultaneously perform cyclic stretching and electrical stimulation. The results of single stimulation showed that either cyclic stretching or electrical stimulation upregulated myogenic gene expression and promoted myotube formation, where electrical stimulation improved better than cyclic stretching. However, only cyclic stretching can align C2C12 cells perpendicular to the stretching direction, and electrical stimulation did not affect cell morphology. Myosin heavy chain (MHC) immunostaining demonstrated that oriented cells under cyclic stretching resulted in parallel myotubes. The combination of these two stimuli exhibited synergetic effects on both myogenic gene regulation and myotube formation, and the incorporated electrical field did not affect the orientation effect of the cyclic stretching. These results suggested that these two treatments likely influenced cells through different pathways. Overall, the simultaneous application of cyclic stretching and electrical stimulation preserved both stimuli's advantages, so myo‐differentiation can be highly improved to obtain abundant parallel myotubes, suggesting that our developed multifunctional bioreactor should benefit muscle tissue engineering applications.

**Translational Impact Statement**

A highly stretchable PPy/PDMS membrane was developed to construct a multifunctional bioreactor, which can mechanically and electrically stimulate C2C12 myoblasts. Both stimuli can upregulate myogenic genes of treated cells, so myo‐differentiation and myotube formation were highly promoted. Furthermore, cyclic stretching can align C2C12 myoblasts, so the formed myotubes were parallel. The combination of these two stimuli exhibited synergetic effects, suggesting the potential of this bioreactor for muscle tissue engineering applications.

## 1 INTRODUCTION

Skeletal muscle is the largest organ and represents 40% of the body weight.^1^ Injury causes severe muscle or volumetric muscle loss (VML), which requires muscle transplants, whereas autograft may cause donor‐site morbidity, and allograft is restricted by the limited donor source.^2^ On the other hand, preclinical drug discovery for treating muscle disease needs to be validated by animal models. Considering the high cost of the disease animal models, it is infeasible to screen all drug candidates by animal test directly. To overcome these difficulties, skeletal muscle tissue engineering is developed. Engineered muscle tissue can be constructed by harvesting and amplifying stem or progenitor cells in the lab. Cell differentiation can be induced by providing an appropriate environment to obtain mature muscle tissues.^3^ In addition to biological signals, external stimuli can mimic the physical environment to further accelerate cell differentiation and promote mature tissue formation. Therefore, various physical stimuli have been investigated to determine their effects on myogenesis facilitation. Because muscle tissues are in charge of physical activity, they are sensitive to environmental mechanical cues.^4^ For example, cyclic stretching can deform cells and this mechanical signal not only promotes cytoskeleton remodeling but also manipulates gene regulation.^5^ On the other hand, intracellular calcium signaling highly impact myogenesis because the differentiation of myoblasts can be inhibited by blocking calcium channels.^6^ Therefore, electrical stimulation is frequently used for muscle tissue engineering because it can alter membrane potential to trigger calcium influx through voltage‐gated L‐type calcium channels. Elevated levels of intracellular calcium ions can improve the differentiation of myoblasts, which not only accelerates the assembly of sarcomeres but also supports muscle maturation.^7^

Due to the promotion effects of cyclic stretching and electrical stimulation on myo‐differentiation, the development of bioreactors to enable their applications in muscle tissue engineering is necessary. Cells grown on a flexible substrate can be cyclically stretched by mechanical traction. Although mechanical traction can delicately manipulate tensile deformation, these devices are bulky and costly and can only process a limited number of samples each time.^8^, ^9^, ^10^ In contrast, vacuum pressure may also stretch flexible membranes.^11^, ^12^ Because vacuum pressure only needs an air pipe connection, it can save device volume and is suitable for use in ordinary incubators. In addition, multiple devices can also be operated simultaneously by connecting air pipes, suggesting its potential for general application. Regarding electrical stimulation, the insertion of electrodes in a culture medium is commonly used to electrically treat cells.^13^ Although this fluid‐mediated method can be broadly applied to different culture environments, electrochemical reactions may occur to interfere with cell physiology.^14^ In addition, electrode corrosion and the corresponding pH change may also be cytotoxic.^15^ Considering that the electrical signal in the physiological environment is transmitted through the tissues, electrical stimulation through a conductive substrate should be more biomimetic.^16^

Different bioreactors have been fabricated based on the abovementioned strategies. However, most of them are designed for a single treatment, so combining multiple stimulations for muscle tissue engineering applications is a challenge. Interactions between various stimuli are also interesting because they probably affect cell physiology through different pathways, and their simultaneous administration may exhibit synergetic or interfering effects. Furthermore, cyclic stretching and electrical stimulation are both directional stimuli, and thus changing their relative directions perhaps causes different influences. Therefore, it is essential to develop a multifunctional bioreactor to comprehensively evaluate the combinational effects of cyclic stretching and electrical stimulation on myogenesis.

**FIGURE 1:**
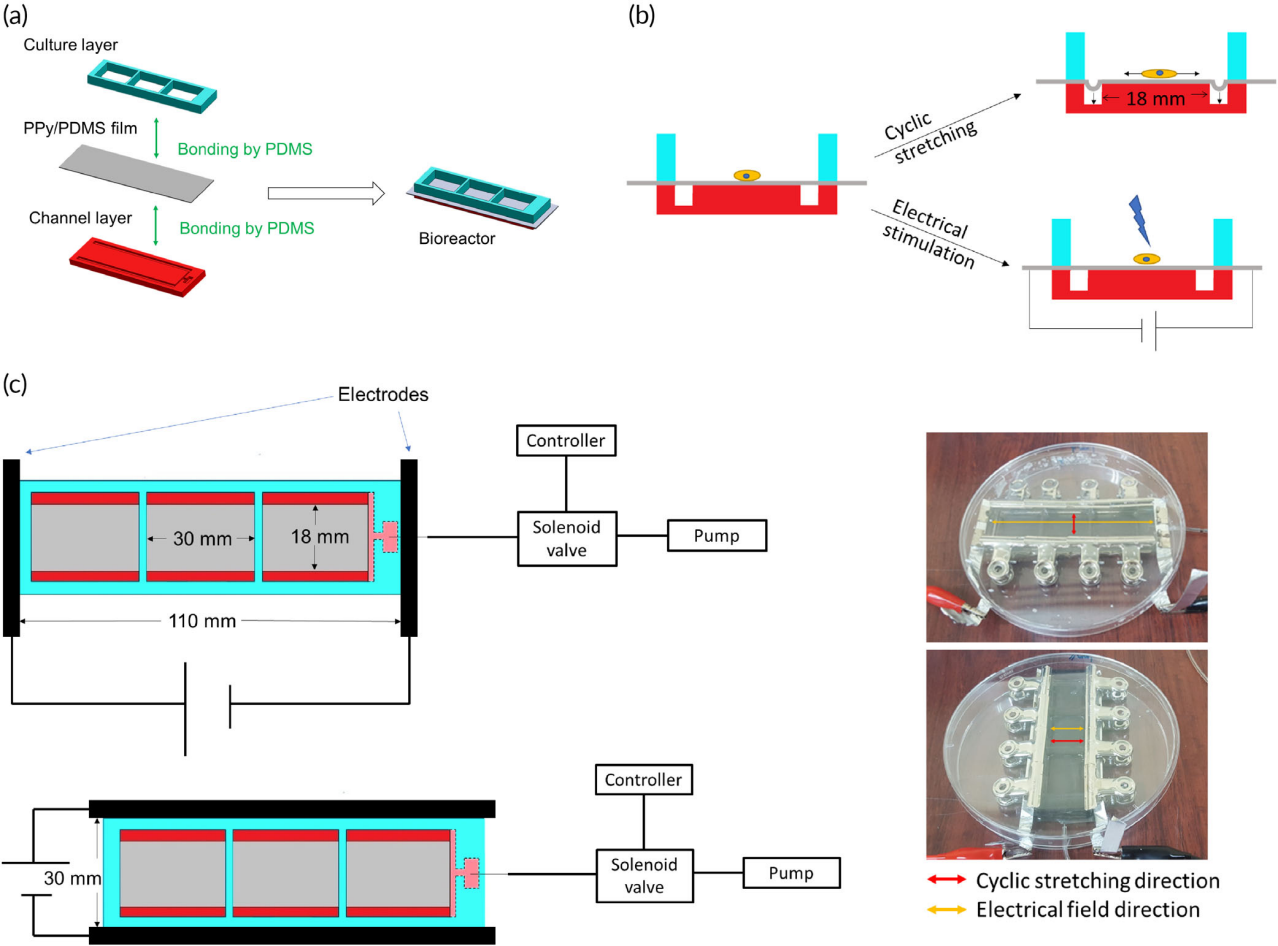
The construction of a multifunctional bioreactor for electrical and mechanical treatments. (a) The bioreactor was assembled as a three‐layered construct, and polydimethylsiloxane (PDMS) was applied to glue between layers. (b) Because polypyrrole (PPy)/PDMS is flexible with high conductivity, cells that adhere to PPy/PDMS membranes can either be cyclic stretched through a vacuum or electrically stimulated by an external electrical field. (c) To investigate the direction effects of electrical and mechanical treatments, two electrodes were connected to two opposite sites either perpendicular (top) or parallel (bottom) to the grooves of the channel layers, so the direction of cyclical stretching and electrical stimulations may thus be perpendicular or parallel to each other, respectively.

In this study, we deposited polypyrrole (PPy) on the surface of polydimethylsiloxane (PDMS) to obtain a highly stretchable conductive PPy/PDMS membrane. Because this PPy/PDMS membrane has excellent light transmittance, cells that adhere to its surface can be directly monitored. Therefore, we applied it to construct a bioreactor (Figure 1). The PPy/PDMS membrane can be stretched rapidly at an arbitrary frequency by regulating vacuum pressure. It can be used to individually or simultaneously apply cyclic stretching and electrical fields to stimulate cells. Therefore, we first optimized these two treatments to myoblasts and then investigated their interaction, including their direction effects and whether these two treatments demonstrated a synergism. By analyzing gene regulation and myotube formation, we may evaluate the potential of applying cyclic stretching and electrical stimulation to promote muscle tissue regeneration.

## 2 RESULTS

### 2.1 The characteristics of bioreactor

**FIGURE 2:**
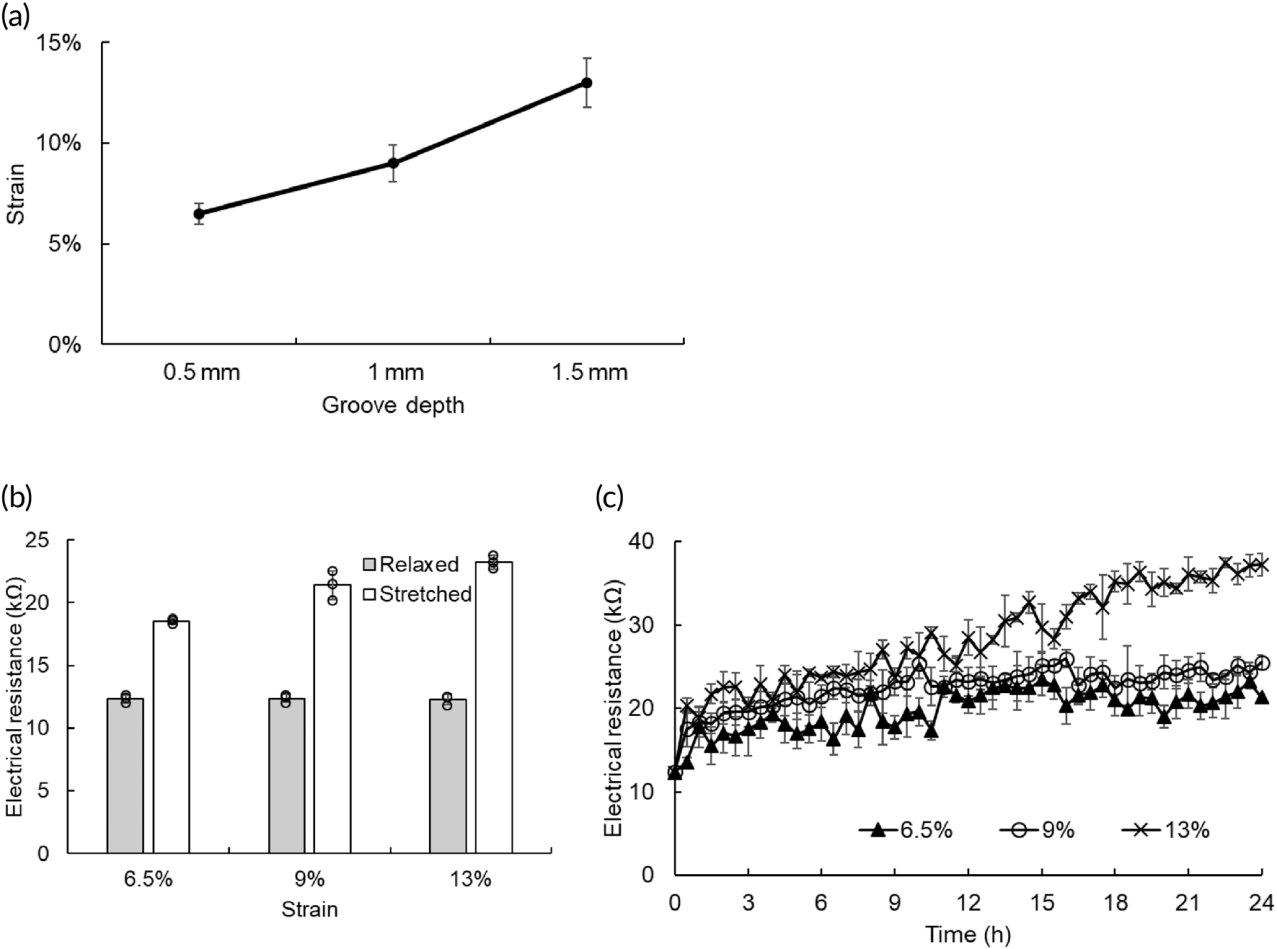
Application of polypyrrole (PPy)/polydimethylsiloxane (PDMS) in cyclic stretching and its conductivity. (a) Channels in grooves in depth of 0.5, 1, and 1.5 mm were used to construct the bioreactor, and the resulting tensile strains were 6.5%, 9%, and 13%, respectively. (b) The electrical resistance of unstretched PPy/PDMS was 12 kΩ. When the membrane was stretched, the electrical resistance was increased to 19–23 kΩ, and higher tensile strains resulted in greater resistances. (c) The electrical resistances under cyclic stretching in different strain were evaluated for 24 h. Although the electrical resistances slightly increased over time, the electrical resistance can be maintained lower than 40 kΩ, suggesting the reliability of conductive PPy/PDMS during cyclic stretching experiments.

Tensile strains generated by vacuum highly depended on the depth of grooves in the channel layer, so we used a microcontact printing technique to track the movement of fluorescent dots due to vacuum‐caused membrane deformation (Figure S1). Nine regions in the wells were monitored to confirm whether the deformation was evenly distributed (Figure S1a), and the tensile strains were determined by comparing the distance between dots with and without stretching (Figure S1b). Our results showed that the deformation was uniform in the well, and the deeper grooves led to the greater deformation. The grooves in depths of 0.5, 1, and 1.5 mm caused tensile strains of 6.5%, 9%, and 13%, respectively (Figure 2a). The surface of PPy/PDMS was intact after cyclic stretching for 4 h, suggesting its reliability in repeated stretching (Figure S1c).

To investigate the effect of stretching on electrical stimulation experiments, the electrical resistances of PPy/PDMS with and without stretching were evaluated (Figure 2b). The relaxed PPy/PDMS demonstrated an electrical resistance of 12 kΩ. When the membrane was stretched, electrical resistances slightly increased with tensile strain, ~18–23 kΩ. Because this bioreactor was designed to perform both cyclic stretching and electrical stimulation, the stability of conductivity performance is impermanent. Therefore, the PPy/PDMS was repeatedly stretched for 24 h and the change in the resistance was monitored (Figure 2c). For the groups using 6.5% and 9% strains, their resistances were stable after 24 h of cyclic stretching. When the strain increased to 13%, the resistance increased slightly over time, whereas it remained lower than 40 kΩ after 24 h of cyclic stretching. These results indicated that the conductive PPy layer was intact under cyclic stretching, and thus the PPy/PDMS membrane was suitable to perform both electrical and mechanical stimulations.

### 2.2 The effects of electrical stimulation on C2C12 cells

**FIGURE 3:**
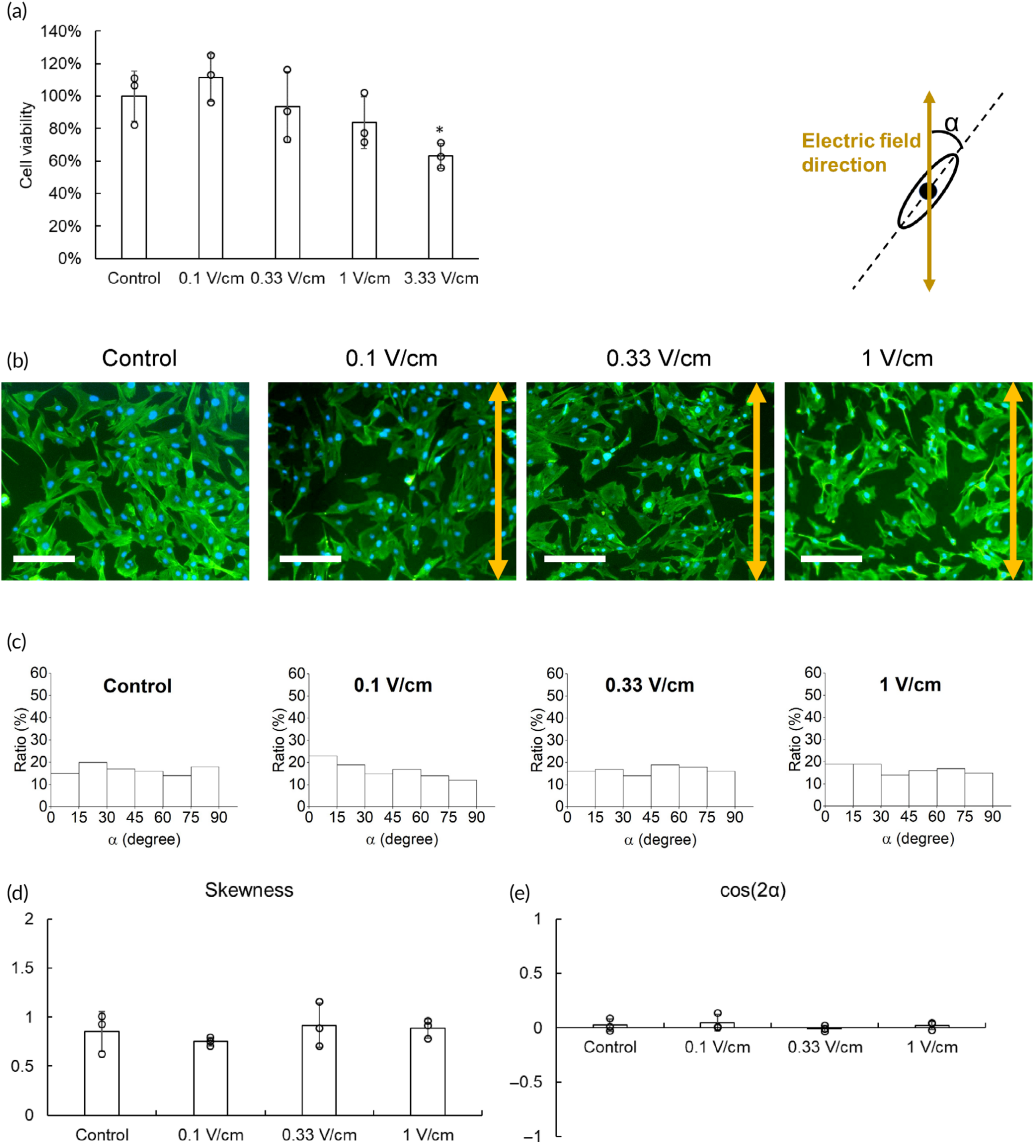
The effect of electrical stimulation on cell viability and morphology. (a) Direct current (DC) in different electric fields was used to treat C2C12 cells 4 h a day for 4 days. The 3‐(4,5‐dimethylthiazol‐2‐yl)‐2,5‐diphenyltetrazolium bromide assay evaluated the viability of treated cells, and the results were relatively expressed by comparing to the control group which was only C2C12 cells in the reactor without electrical treatment. (b) The morphology of C2C12 cells was examined by fluorescent staining. The direction of the electric field was indicated as a yellow double arrow (green: phalloidin‐FITC for cytoskeleton, blue: DAPI for nuclei) (scale bar = 100 μm). (c) Through these fluorescent images, the orientation of treated cells was determined by α which was the angle between the long axis of the cell and the electric field direction. The (d) skewness and (e) average cos(2α) were also determined.

Direct current (DC) was applied as an electrical stimulation to treat C2C12 cells 4 h a day for 4 days. These viabilities of treated cells were evaluated by a 3‐(4,5‐dimethylthiazol‐2‐yl)‐2,5‐diphenyltetrazolium bromide (MTT) test (Figure 3a). Although the lowest electric field (0.1 V/cm) slightly improved the MTT value, the viability of treated cells became reduced when the electric field kept rising. The highest electric field group (3.33 V/cm) even demonstrated significantly lower viability than the control group, suggesting excessive electrical stimulation may harm the cells. Therefore, we only used electric fields in the 0.1–1 V/cm range in the following experiments.

The morphology examination showed that C2C12 cells with and without electrical stimulation all exhibited a radial branching morphology (Figure 3b). These cell images were analyzed by Image J to determine the angle α between the long axis of cells and the direction of the electric field (Figure 3c), and these results were analyzed to assess skewness and cos (2α) (Figure 3d,e). Regardless of whether the cells were subjected to electrical treatment, they demonstrated skewness and cos(2α) values close to 1 and 0, respectively, suggesting that the C2C12 cells exhibited isotropic extension and electrical stimulation should not affect cell morphology.

**FIGURE 4:**
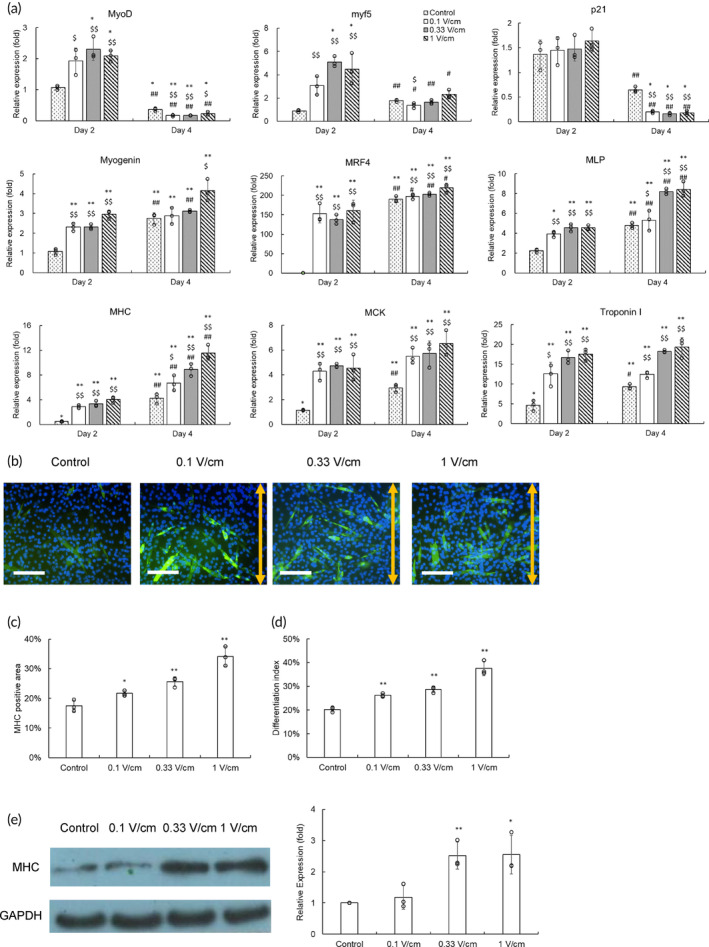
The effects of electrical stimulation on gene regulation of differentiation C2C12 cells and myotube formation. (a) After changing to myogenic medium, electrical stimulation was applied to treat C2C12 cells 4 h a day for 4 days. The control group was C1C12 cells in the bioreactor without treatment. The mRNA of C2C12 cells was harvested and evaluated by a quantitative polymerase chain reaction assay to determine gene regulation. The results were expressed as relative levels by comparing with those from the cells on Day 0 in the bioreactor (*p < 0.05, **p < 0.01 compared with the control group on Day 0; $*p* < 0.05, $$*p* < 0.01 compared with the control group on the same day; #*p* < 0.05, ##*p* < 0.01 compared with the corresponding results on Day 2). (b) Immunostaining of myosin heavy chain (MHC, green) was applied to illustrate myotubes, and their nuclei were stained by DAPI (blue). The electrical field direction was indicated as a yellow double arrow (scale bar = 100 μm). (c) The MHC stained area in the fluorescent images was quantified by the image software. (d) The proportion of nuclei in fused myotubes was expressed as the differentiation index. (e) The western blotting analysis of MHC. The quantitative results of MHC were normalized by the corresponding GAPDH and expressed as relative levels by comparing with the control group (*: *p* < 0.05, **: *p* < 0.01 compared with the control group).

Then, we evaluated the effect of electrical stimulation on myogenic differentiation. Differentiation of C2C12 cells was triggered by applying myogenic medium for 4 days and concurrently with electrical stimulation treatment. Quantitative polymerase chain reaction (qPCR) was applied to evaluate gene regulation, and the results were expressed as relative levels by comparing them to the results from the cells on Day 0 in the bioreactor (Figure 4a).

Both myf5 and MyoD are myogenic regulatory factors that activate both themselves and other myogenic regulatory factors.^17^ They are upstream markers and are involved in initiating C2C12 differentiation. Therefore, *MyoD* expression increased on Day 2, then decreased on Day 4, and electrical stimulation significantly enhanced *MyoD* expression on Day 2. A similar trend was found in *myf5* expression. Interestingly, the upregulation of *myf5* in cells under electrical stimulation was on Day 2, whereas in the untreated cells was on Day 4. It indicated that electrical stimulation accelerated *myf5* upregulation. On the other hand, p21 is a cyclin‐dependent kinase inhibitor that induces cell cycle arrest to go to the differentiation pathway.^18^ Therefore, the levels of *p21* also increased and then decreased during C2C12 differentiation. Because the decrease trends of electrical stimulation groups were more evident than the control group, the improvement effect of electrical stimulation may be related to cell cycle arrest.

Myogenin induces the fusion of myoblasts to form myotubes.^19^ MRF4 further matures and hypertrophies myotubes.^20^ MLP acts as a cofactor by binding MyoD, myogenin, and MRF4 to enhance their activity.^21^ Therefore, *Myogenin*, *MRF4*, and *MLP* are typical markers in the mid‐last stage of myogenic differentiation. *MHC*, *MCK*, and *Troponin I* are genes related to myotube structure. Myosin heavy chain (MHC) is the major contractile protein in muscle tissue. Muscle creatine kinase (MCK) and Troponin I are related to muscle contraction because MCK provides ATP in muscle cells and Troponin I binds actin to stabilize the actin–tropomyosin complex.^22^, ^23^ Consequently, we investigated these genes to determine the effects of stimulation on promoting myotubes formation and maturation. Regarding the control group, the levels of *Myogenin*, *MRF4*, *MHC*, and *MCK* on Day 2 were almost the same as those before treating the myogenic medium, and their *MLP* and *Troponin I* only slightly increased. The upregulation of these genes was mainly on Day 4. In contrast, electrical stimulation highly upregulated the expression levels of all genes on Day 2 and further improved on Day 4. The improvement was dose‐dependent and the group of 1 V/cm demonstrated the highest gene expression. These results indicated that electrical stimulation likely accelerated myogenic differentiation.

Immunostaining of MHC was applied to evaluate myotube formation (Figure 4b). The electrical treatment seemed to lead C2C12 cells to form more myotubes than those without stimulation. Furthermore, because electrical stimulation did not manipulate the morphology of C2C12 cells (Figure 3b), myotubes formed under electrical stimulation were randomly orientated, just like the control group. These fluorescent images were evaluated by the image software and the results suggested that electrical stimulation indeed promoted MHC expression and this improvement directly depended on the electric field (Figure 4c). The differentiation index showed that electrical stimulation also enhanced the ratio of differentiated cells (Figure 4d). Western blot assay showed that an electrical field equal to or over 0.33 V/cm significantly increased MHC expression (Figure 4e). These results indicated that electrical stimulation effectively improved myogenic differentiation and myotube formation.

### 2.3 The effects of cyclic stretching on C2C12 cells

**FIGURE 5:**
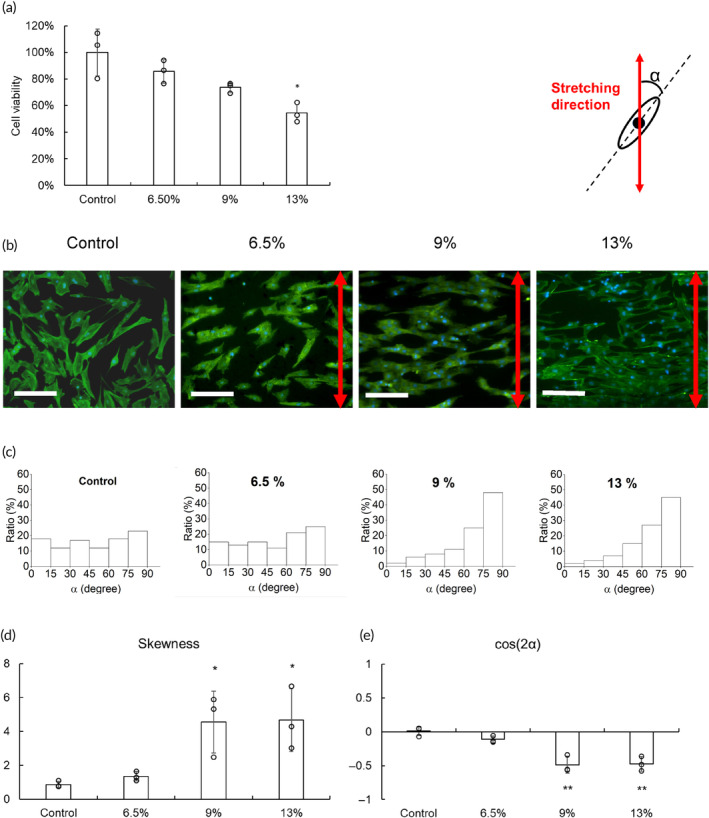
The effect of cyclic stretching on cell viability and morphology. (a) Cyclic stretching in different tensile strains was used to treat C2C12 cells 6 h a day for 4 days. The 3‐(4,5‐dimethylthiazol‐2‐yl)‐2,5‐diphenyltetrazolium bromide assay evaluated the viability of treated cells, and the results were relatively expressed by comparing to the control group which was only C2C12 cells in the reactor without stretching treatment. (b) The morphology of C2C12 cells was examined by fluorescent staining. The direction of stretching was indicated as a red double arrow (green: phalloidin‐FITC for cytoskeleton, blue: DAPI for nuclei; scale bar = 100 μm). (c) Through these fluorescent images, the orientation of treated cells was determined by α which was the angle between the long axis of the cell and the electric field direction. The (d) skewness and (e) average cos(2α) were also determined (*p < 0.05, **p < 0.01 compared with the control group).

A pulsatile vacuum in the frequency of 1 Hz was applied to stretch PPy/PDMS, by which C2C12 cells were cyclically stretched. These cells were stimulated 6 h a day for 4 days. The viability of C2C12 cells under cyclic stretching was examined by the MTT test (Figure 5a). Compared with the control group, cyclic stretching reduced cell viability, most notably in the 13% strain group. Cell morphology was then examined (Figure 5b). Like the control group, C2C12 cells stretched by 6.5% strain demonstrated a radial branching morphology. In contrast, cells under 9% and 13% strains were relatively extended, and their orientation distribution showed that more than 70% of the cells in these two groups exhibited an angle *α* >60° (Figure 5c). In addition, the skewness of the 9% and 13% groups were higher than 4 and their cos (2α) were higher than −0.5, suggesting that cyclic stretching in a tensile strain equal to or over 9% may cause cell extension mainly perpendicular to the stretching direction (Figure 5d,e). These results indicated that cyclic stretching can manipulate cell morphology and align them with each other.

**FIGURE 6:**
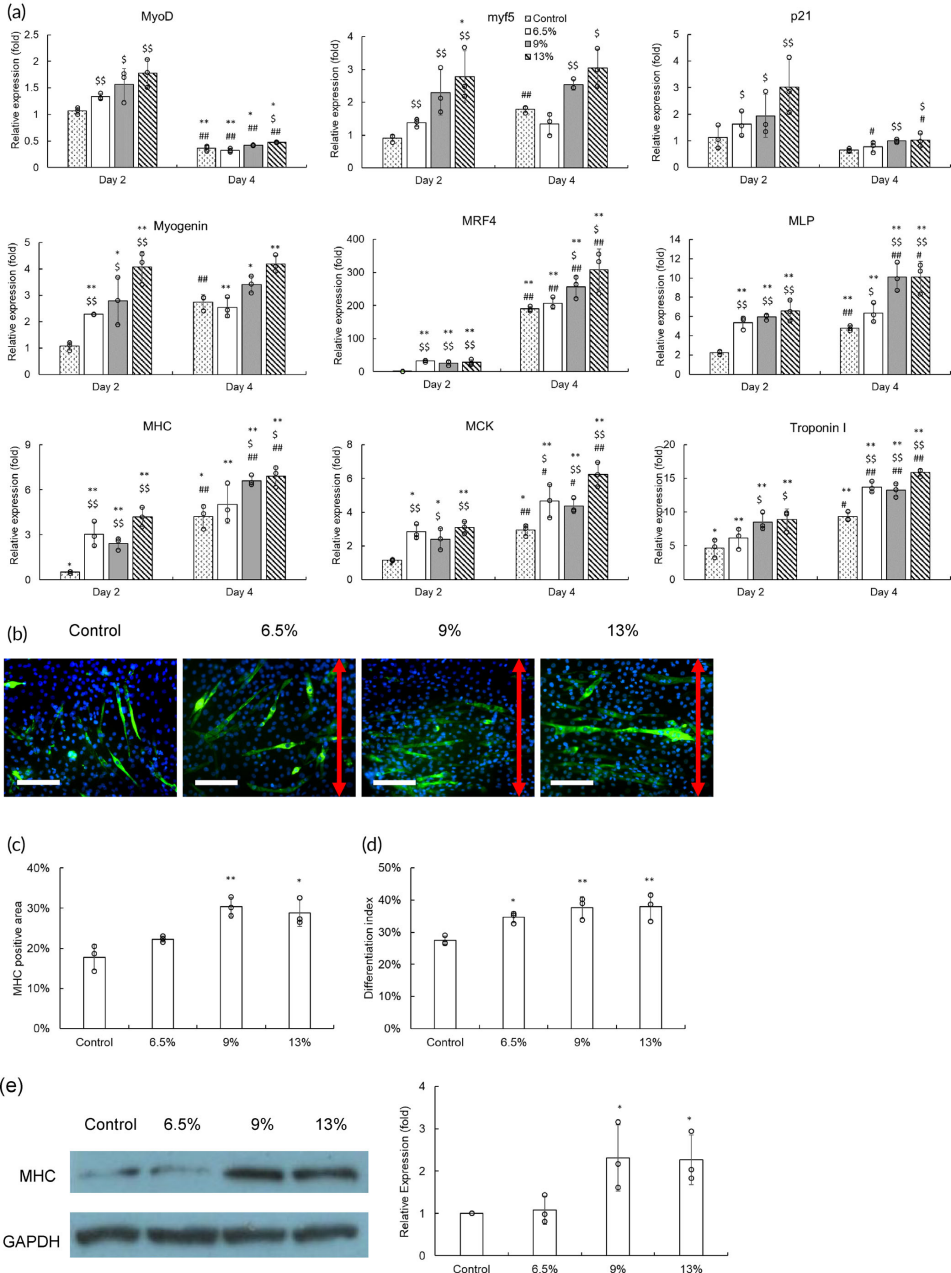
The effects of cyclic stretching on gene regulation of differentiation C2C12 cells and myotube formation. (a) After changing to a myogenic medium, cyclic stretching was applied to treat C2C12 cells 6 h a day for 4 days. The control group was C1C12 cells in the bioreactor without treatment. The mRNA of C2C12 cells was harvested and evaluated by a qPCR assay to determine gene regulation. The results were expressed as relative levels by comparing with those from the cells on Day 0 in the bioreactor (*p < 0.05, **p < 0.01 compared with the control group on Day 0; $*p* < 0.05, $$*p* < 0.01 compared with the control group on the same day; #*p* < 0.05, ##*p* < 0.01 compared with the corresponding results on Day 2) (b) Immunostaining of myosin heavy chain (MHC) (green) was applied to illustrate myotubes, and their nuclei were stained by DAPI (blue). The electrical field direction was indicated as a red double arrow (scale bar = 100 μm). (c) The imaging software quantified the MHC stained area in the fluorescent images. (d) The proportion of nuclei in fused myotubes was expressed as the differentiation index. (e) The western blotting analysis of MHC. The quantitative results of MHC were normalized by the corresponding GAPDH and expressed as relative levels by comparing with the control group (*p < 0.05, **p < 0.01 compared with the control group).

The effects of cyclic stretching on myogenic differentiation were then evaluated. Differentiation of C2C12 cells was triggered by applying myogenic medium for 4 days and concurrently with cyclic stretching. The gene regulation was evaluated by the qPCR analysis (Figure 6a).

The control group's *MyoD* and *myf5* levels remained unchanged on Day 2, and only *myf5* increased on Day 4. In contrast, cyclic stretching promoted these two upstream markers on Day 2 in a dose‐dependent behavior, suggesting that cyclic stretching may accelerate differentiation. Different from the electrical stimulation which promoted *p21* down‐regulation on Day 4, the effect of cyclic stretching was mainly on upregulating *p21* on Day 2, so we deduced that cyclic stretching probably also affected the cell cycle but through a mechanism different from that of the electrical stimulation.

For the myogenic makers of the mid‐last differentiation stage, cyclic stretching allowed cells to increase *Myogenin*, *MRF4*, and *MLP* levels on Day 2, whereas the control group did not upregulate these genes until Day 4. The genes relative to myotubes, that is, *MHC*, *MCK*, and *Troponin I*, also demonstrated the same trend. Furthermore, the promotion effects of cyclic stretching increased with the tensile strain.

The immunostaining of MHC showed that myotubes formed at 6.5% cyclic tensile strain were randomly oriented, just like the control group (Figure 6b). In contrast, the 9% and 13% groups both demonstrated parallel myotubes perpendicular to the stretching direction. This trend was consistent with the cell morphology, suggesting that cell alignment by cyclic stretching can facilitate parallel myotube formation. In addition, the quantification also indicated that cyclic stretching not only increased myotubes but also promoted differentiation levels (Figure 6c–e).

### 2.4 The combination of electrical stimulation and cyclic stretching

**FIGURE 7:**
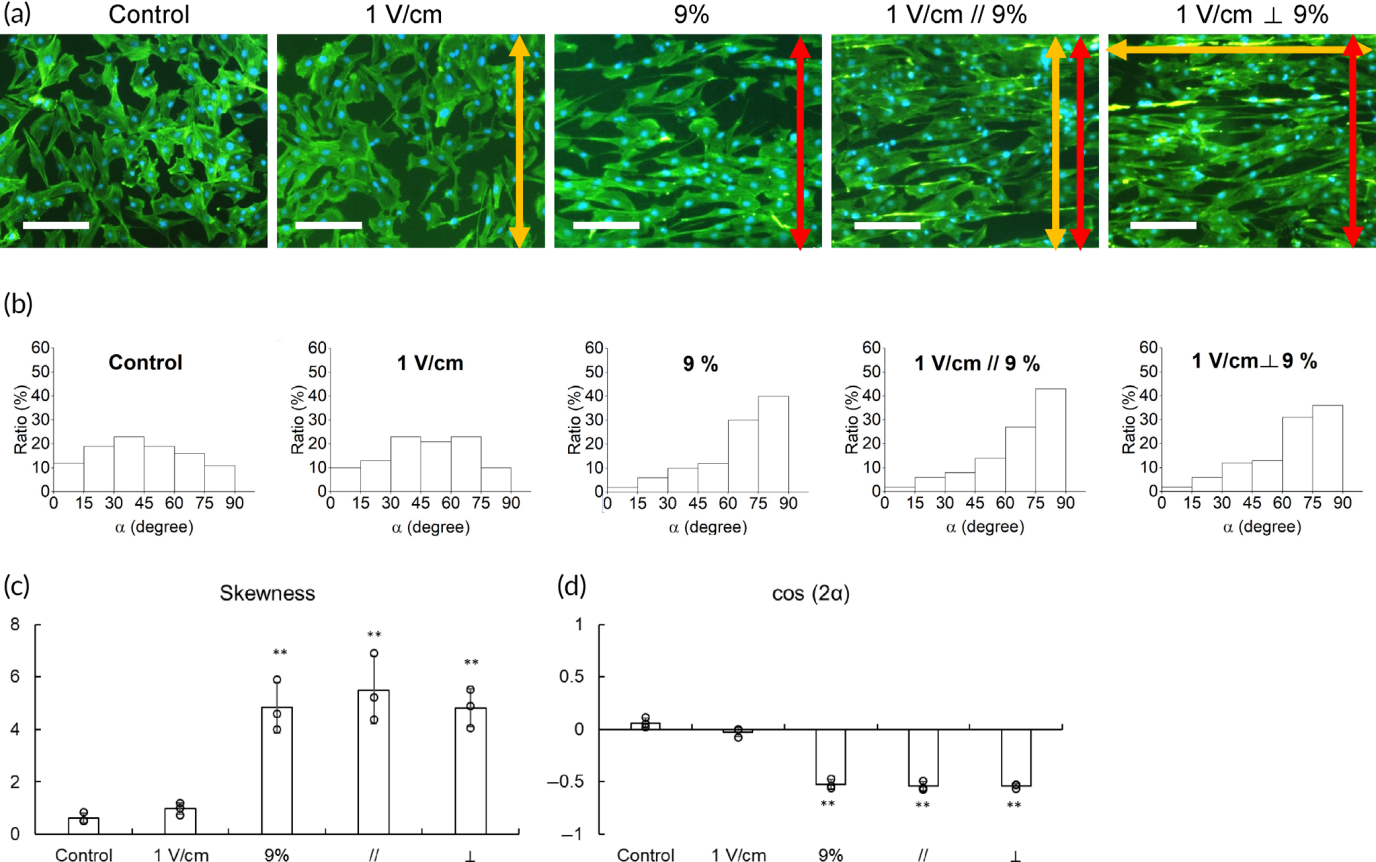
The morphology of cells under multiple stimulations. (a) Morphology of C2C12 cells was examined by fluorescent staining. The stretching directions and electric field direction were indicated by red and yellow double arrows, respectively (green: phalloidin‐FITC for cytoskeleton, blue: DAPI for nuclei; scale bar = 100 μm). (b) Through these fluorescent images, the orientation of treated cells was determined by α which was the angle between the long axis of the cell and the stretching direction. The (c) skewness and (d) average cos(2α) were also determined (*p < 0.05, **p < 0.01 compared with the control group).

Finally, we further investigated the combination effects of these two stimulations. Cyclic stretching and electrical stimulation were both applied to treat C2C12 cells for 4 days, and the results were compared with those of cyclic stretching or electrical stimulation alone. To determine the direction effects of the combination experiments, the stretching direction was either parallel or perpendicular to the direction of the electric field. We used 9% tensile strain for cyclic stretching and 1 V/cm of electric field for electrical stimulation due to their optimal performances in prior experiments. The morphology results showed that the dual stimulation groups in parallel or perpendicular directions both aligned cells perpendicular to the stretching direction (Figure 7a). To determine the level of orientation, the angle α between the long axis of the cell and the stretching direction was evaluated (Figure 7b). Parallel or perpendicular dual stimulation groups both demonstrated skewness between 4 and 5 and cos 2α close to −0.6, which were almost the same as that of the only cyclic stretching group. It suggested that electrical stimulation did not alter the alignment effect of the cyclic stretching (Figure 7c,d).

**FIGURE 8:**
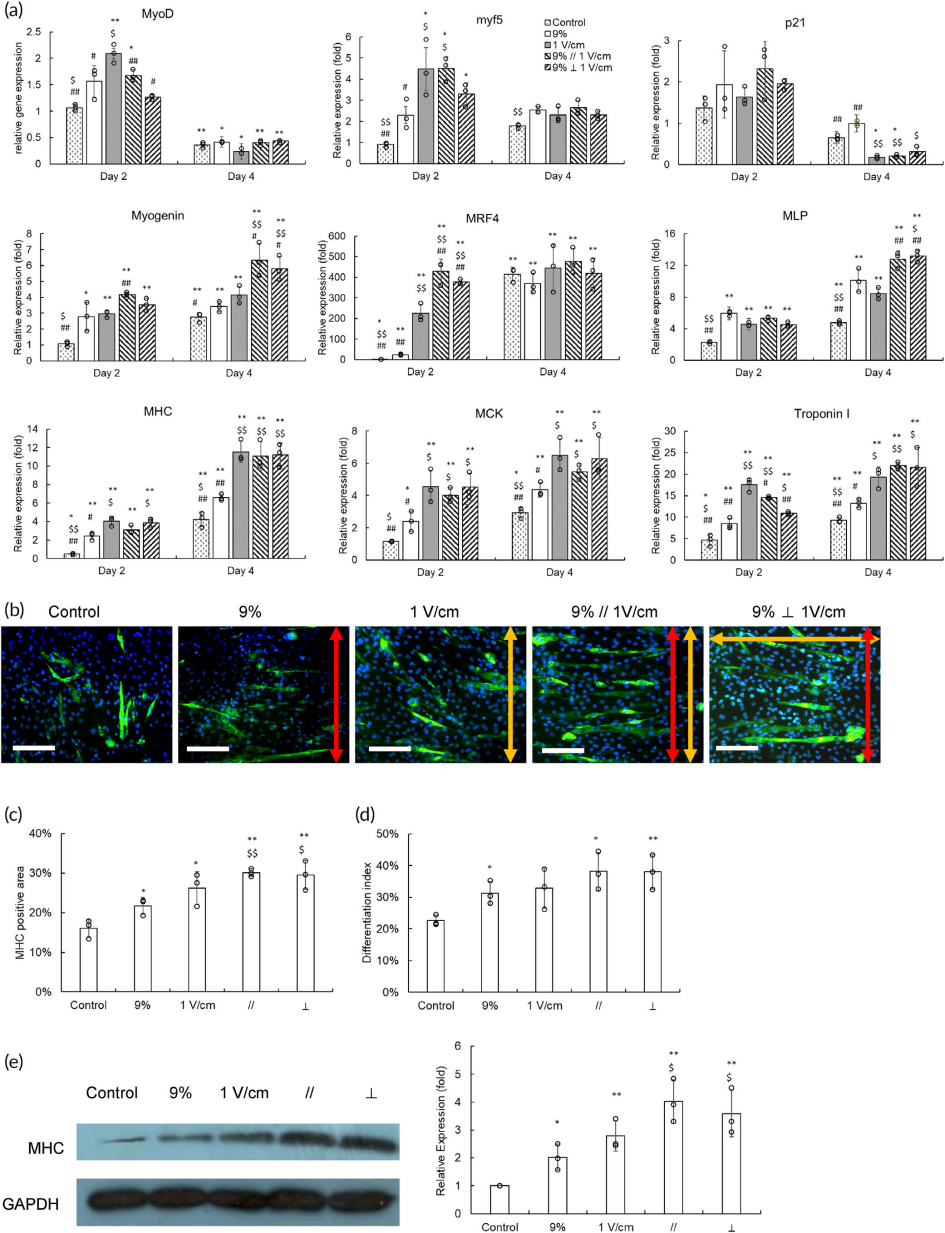
The effects of combinational stimulations on gene regulation of differentiation C2C12 cells and myotube formation. (a) After changing to a myogenic medium, C2C12 cells were treated with cyclic stretching and electrical stimulation, individually or in combination, for 4 days. The control group was C1C12 cells in the bioreactor without treatment. The mRNA of C2C12 cells was harvested and evaluated by a quantitative polymerase chain reaction assay to determine gene regulation. The results were expressed as relative levels by comparing with those from the cells on Day 0 in the bioreactor (*p < 0.05, **p < 0.01 compared with the control group on Day 0; $*p* < 0.05, $$*p* < 0.01 compared with the 9% group on the same day; #*p* < 0.05, ##*p* < 0.01 compared with the 1 V/cm group on the same day). (b) Immunostaining of myosin heavy chain (MHC, green) was applied to illustrate myotubes, and their nuclei were stained by DAPI (blue). The stretching directions and electric field direction were indicated by red and yellow double arrows, respectively (scale bar = 100 μm). (c) The imaging software quantified the MHC stained area in the fluorescent images. (d) The proportion of nuclei in fused myotubes was expressed as the differentiation index. (e) The western blotting analysis of MHC. The quantitative results of MHC were normalized by the corresponding GAPDH and expressed as relative levels by comparing with the control group (*p < 0.05, **p < 0.01 compared with the control group; $*p* < 0.05, $$*p* < 0.01 compared with the 9% group).

Then, we applied qPCR analysis for gene regulation evaluation (Figure 8a). Regarding single stimuli, electrical stimulation seemed to cause better promotion effects than cyclic stretching, such as *MyoD*, *myf5*, *MRF4*, *MHC*, *MCK*, and *Troponin I* on Day 2 and *MHC*, *MCK*, and *Troponin I* on Day 4. Dual stimulation exhibited a similar or even better promotion effect than these single stimulations. For example, the upregulation of *MRF4* of the dual stimulation on Day 2 was greater than the cyclic stretching and electrical stimulation groups, and *Myogenin* and *MLP* on Day 4 also demonstrated the same trend. The effects of physical stimulation on gene regulation are illustrated in a scheme (Figure S2).

Regarding myotube formation, the MHC staining results indicated that dual stimulation groups formed myotubes perpendicular to the stretching direction (Figure 8b), consistent with their cell morphology results (Figure 7a). The quantification results showed that electrical stimulation likely increased MHC more efficiently than cyclic stretching, although the difference was insignificant. In contrast, dual stimulation significantly promoted MHC expression compared with the cyclic stretching group, and the western blot assay also demonstrated the same trend (Figure 8c,e). Furthermore, dual stimulation groups demonstrated superior differentiation indies (Figure 8d). These results suggested that combining cyclic stretching and electrical stimulation may synergistically facilitate myogenic differentiation.

## 3 DISCUSSION

In our previous study, we successfully deposited PPy by in situ polymerization of pyrrole in a concentration of 0.5 M, and our results showed that the fabricated PPy/PDMS membrane demonstrated high conductivity and low cell cytotoxicity, so we applied it to manufacture a bioreactor. This bioreactor can manipulate tensile strain by adjusting groove depth, and the conductive PPy layer allows electric current passthrough to electrically stimulate surface cells.^24^ Although this device owns a large area for cell growth, the single‐well design cannot perform multiple experiments at the same time. In addition, a syringe pump controls the vacuum pressure for membrane stretching. However, mechanically controlled plunger movement restricts the speed of membrane stretching. To overcome these difficulties, we designed a multiwell bioreactor in this study that can be used for triplicate experiments. A vacuum pump was applied to stretch the membrane rapidly, and a timer‐controlled solenoid valve could regulate the stretching frequency. By connecting electrodes on two opposite sides of the membrane, this bioreactor can perform both cyclic stretching and electrical stimulation.

Stress fibers are bundles of contractile actin fibers and play an essential role in cell morphology due to their contractility. Because cyclic stretching may disrupt stress fibers, the distribution of stress fibers is inclined to the direction of the least tensile strain, that is, the direction perpendicular to the stress.^25^ Therefore, cells under cyclic stretching are vertically extended to the stretching direction, which is called strain avoidance response.^26^ Although cells tend to reorient to reduce stress damage, it is difficult to ideally rearrange their all morphology due to the limited space occupied by neighboring cells. Therefore, the strain avoidance response cannot ideally make all cells align perpendicular to the stretching direction. When some cells fail to orient their stress fibers toward the direction of minimal stress, these stress fibers are destroyed and inevitably result in the collapse of the cytoskeleton and cell death.^27^ We have used a channel layer in a groove depth of 2 mm to stretch the membrane, which can result in a strain of 17%. We found that when cells were cyclically stretched in a strain of 17%, they would detach from PPy/PDMS after 4 h treatment (data now shown). Our MTT results also demonstrated that cyclic stretching gradually decreased cellular viability with increasing strains, and the 13% strain group even reduced to <60% compared with the untreated group, suggesting that excessive stretching is harmful.

Although overstretching may cause cell damage, appropriate cyclic stretching can align cells in parallel and preserve their bioactivity. In addition to cell alignment, our results also indicated that cyclic stretching facilitated myogenic differentiation and tube formation. Pennisi et al.^28^ applied 0.5 Hz of cyclic stretching in a tensile strain of 15% to treat C2C12 cells and used immunostaining to label expressed myogenin and MHC. Compared with the untreated cells, cyclic stretching can boost the expression of myogenin expression on Day 2, so the MHC expression was highly expressed in the mature myotubes on Day 5. Due to the early expression of myogenin, Pennisi suggested that cyclic stretching may accelerate myogenic differentiation.

Different types of electrical signals have been studied to treat muscle tissue. Pulsatile currents are broadly used to stimulate cardiomyocytes due to the importance of functional synchronization. Therefore, cardiac cells' alignment, electrical coupling, and phenotype maintenance are highly improved under pulsatile currents.^29^, ^30^ Electrical pulse stimulation has also been reported to enhance the proliferation and differentiation of skeletal myoblasts.^7^, ^31^ Relatively few studies apply DC to treat myoblasts. Nevertheless, some research indicates that DC is important in embryonic development because the voltage gradient can guide cell migration and manipulate cell morphology.^32^, ^33^, ^34^ Therefore, we decided to use DC to treat C2C12 cells and evaluated whether DC can manipulate cell morphology and promote myogenic differentiation. Through connecting two ends of PPy/PDMS to electrodes, C2C12 cells were directly stimulated by passing DC.

The MTT results showed that 0.1 V/cm increased cell bioactivity to 112%; however, bioactivity decreased to 63% when the electrical field increased to 3.33 V/cm (Figure 3a). It has been indicated that electrical stimulation may upregulate the VEGF gene,^35^ and VEGF has been proven to facilitate the proliferation of C2C12 cells.^36^ Therefore, we deduced that the expression of VEGF may be increased by electrical stimulation to promote cell proliferation, whereas cells may be damaged when the voltage is too high.^37^

Our results demonstrated that both cyclic stretching and electrical stimulation could promote myogenic differentiation. However, only cyclic stretching can manipulate cell orientation. It suggests that electrical stimulation in our device should be independent of cell morphology. Currently, placing electrodes in medium or hydrogel is the most frequently used method to electrically stimulate cells. This liquid‐mediate electrical stimulation has been indicated to promote myogenesis and cell alignment. For example, Liu et al.^38^ placed electrodes in a culture medium to electrically treat C2C12 cells, and their results indicated that C2C12 cell alignment and myotube formation are improved when the electrical field is equal to or higher than 1.5 V/cm. These cells probably are stimulated through electrochemical reactions in electrodes or the electrophoresis of ions in the liquid phase when electrical current passes through the conductive medium.^39^, ^40^ Unlike the liquid‐mediated treatment, electrical stimulation in our bioreactor was mediated by the conductive PPy substrate, so redox reactions or ion flows did not occur in our device. Therefore, we deduced that the promotion pathways of the liquid‐mediated and substrate‐mediated electrical stimulations are perhaps different. We also applied a thermal imager to analyze whether PPy/PDMS increased its temperature during electrical stimulation (Figure S3). Our result showed that the temperature of the PPy/PDMS was almost the same as that of the environment, suggesting that our device did not elicit joule heating.

Although cells react and adapt to the chemical and physical signals of the microenvironment is not yet fully understood, mechanotransduction, which involves the reciprocal feedback loop between the outside‐in and inside‐out signaling pathways, is conventionally thought of as the process by which mechanical stimuli are converted into biochemical signals.^41^ Furthermore, mechanical force may be propagated through the nucleus to alter gene expression by linkers of the nucleoskeleton and cytoskeleton (LINC).^42^ For example, to prevent repeated stretching damage to stress fibers, stress fibers deformed by mechanical stimuli would induce the expression of myosin II‐a through the RhoA signal pathway to achieve strain avoidance response.^27^ On the other hand, electrical stimulation is also broadly applied to facilitate myogenesis, support muscle maturation, and accelerate sarcomere assembly to obtain enhanced contractility.^7^ Calcium is an essential biological signal to regulate cellular physiology. The differentiation of myoblasts can be reduced by inhibiting their L‐type calcium channels, supporting that the influx of calcium plays a crucial role in myogenesis.^6^ Considering the fact that the L‐type calcium channels are voltage‐gated, electrical stimulation is a promising strategy to induce calcium influx by adjusting the membrane potential of myoblasts.^7^ The rise of intracellular calcium can trigger intracellular signal pathways, such as PI3K/AKT/mTOR and MAPK/ERK, which highly impact gene regulation and certain protein expression.^43^

Since electrical stimulation and cyclic stretching both improved cell differentiation and cell function, we applied qPCR analysis to elucidate the promotion effects on gene regulation. These two stimuli seemed to exhibit different effects. For *MyoD* and *myf5*, the electrical stimulation and dual stimulation groups all increased on Day 2 and then decreased on Day 4, and their change ranges were large, whereas these two genes were only upregulated modestly in the Day 2 results of the cyclic stretching group. In contrast, *MyoD* and *myf5* of the untreated group were unchanged on Day 2, and *myf5* was increased on Day 4. In addition, the downregulation of *p21* on Day 4 was more pronounced in the electrical stimulation and dual stimulation groups compared with the cyclic stretching and the untreated groups. These phenomena suggested that electrical stimulation and cyclic stretching may regulate gene expression at different speeds and intensities (Figure S4). Electrical stimulation likely prompted cells to enter differentiation faster and more robust, so the amplitude and time of downregulation are larger and earlier.

Because dual stimulation groups applied both stimuli, they can preserve both advantages. Therefore, dual stimulation maintained the superior upregulation as electrical stimulation. Regarding the direction effects of these two stimuli, although the *MyoD* of the perpendicular group was significantly lower than that of the parallel stimulation group on Day 2, their other gene regulations were similar. In addition, dual stimulation in different directions demonstrated similar morphology, MHC expression, and differentiation index, suggesting that the directionality between cyclic stretching and electric field has little effect.

In this study, we successfully developed a bioreactor to apply electrical stimulation and cyclic stretching, and our results showed that these two treatments can promote myotube formation and alignment. Except for skeletal muscle, different tissues such as bones,^44^ nerves,^45^ and cardiac tissues^46^ have been reported to be responsive to electrical and mechanical stimulations. Although the promotion effects of these stimulations have been broadly investigated, it is rare to investigate their synergistic effects. Therefore, our device can be a valuable platform to simultaneously perform dual stimulation, which should benefit tissue engineering applications.

## 4 CONCLUSIONS

We successfully developed a multifunctional bioreactor to perform electrical and mechanical stimulations. This device can rapidly stretch PPy/PDMS membrane with controllable tensile strain. The integrity of the membrane was maintained during repeated stretching to preserve its conductivity. When we applied this bioreactor to treat C2C12 myoblasts, both electrical stimulation and cyclic stretching not only accelerated the upregulation of early markers (*MyoD* and *mfy5*) but also improved the levels of myogenic genes in the mid‐late stages (*Myogenin*, *MRF4*, and *MLP*), so their characteristic genes of myotubes (*MHC*, *MCK*, and *Troponin I*) were eventually improved. Therefore, these two stimuli can improve myogenic differentiation and myotube formation. Interestingly, only cyclic stretching can align cells perpendicular to the stretching direction, whereas electrical stimulation has no function on cell morphology. The combination of electrical stimulation and cyclic stretching also highly upregulated genes relative to myogenic differentiation, and some genes, such as *Myogenin*, *MRF4*, and *MLP*, were even synergistically improved. Furthermore, dual stimulations preserve the advantages of both electrical and mechanical stimulations, so the differentiation of myoblasts can be highly improved to obtain abundant parallel myotubes. These results suggest that our developed multifunctional bioreactor should benefit muscle tissue engineering applications.

## 5 MATERIALS AND METHODS

### 5.1 Materials

Pyrrole and dimethyl sulfoxide (DMSO) were procured from Acros Organics (Thermo Fisher Scientific, Geel, Belgium). Dow Corning (Midland, MI, USA) supplied SYLGARDTM™184. Ammonium persulfate (APS), LiCl, and NaOH were obtained from Showa Kako (Osaka, Japan). Cytiva Hyclone (Cat. SH3003.02, Marlborough, MA, USA) provided Dulbecco's Modified Eagle Medium (DMEM), and Gibco (Thermo Fisher Scientific) supplied fetal bovine serum (FBS), horse serum, and trypsin. Additionally, rhodamine B, insulin from bovine, silicon oil AS 100, bovine serum albumin (BSA), formaldehyde, acetic acid, Triton‐X 100, 4′,6‐diamidine‐2′‐phenylindole dihydrochloride (DAPI), MTT, and phalloidin‐FITC were obtained from Sigma Aldrich (Merck, Darmstadt, Germany). C2C12 mouse myoblasts were obtained from the Bioresource Collection and Research Center (Hsinchu, Taiwan).

### 5.2 The preparation of PPy/PDMS membranes

To create a PDMS membrane, we mixed the A and B reagents of SYLGARD™ 184 in a weight ratio of 10:1 and poured the mixture onto a glass surface. Using a scraper, we dispersed the mixture to a thickness of 200 μm before baking it at 85°C for 6 h.

To facilitate PPy deposition, PDMS was etched by 3N NaOH solution for 6 h at room temperature. After thoroughly rinsing with double distilled water, 0.1 M of APS and 0.5 M of pyrrole in equal volumes were added onto PDMS surfaces for in situ polymerization at 4°C for 20 min. Finally, ultrasonication (DC400, Delta Ultrasonic, New Taipei City, Taiwan) was applied to clean these PPy/PDMS membranes.

### 5.3 The construction of the bioreactor

The PPy/PDMS membrane was applied to fabricate a bioreactor. Here, a bioreactor was constructed by assembling a channel layer, a PPy/PDMS membrane, and a culture‐well layer, which were bound together by PDMS under 85°C baking (Figure 1a). The channel layer was cast from a poly (methyl methacrylate) mold which was fabricated by a computerized numerical control (CNC) lathe (EGX400, Twinsoft Industrial Co, New Taipei City, Taiwan; Figure S5a–c). To perform triplicate experiments, a 3‐well culture layer was fabricated by the CNC lathe, too (Figure S6).

### 5.4 The application of bioreactor for cyclic stretching and electrical stimulation

Because PPy/PDMS is a highly stretchable conductive membrane, it can be used to cyclic stretch or electrically stimulate cells on a bioreactor (Figure 1b). A pin‐attached tube was inserted into the channel layer and connected to a vacuum pump (Rocker 300, Rocker Scientific Co., Kaohsiung, Taiwan) to cause PPy/PDMS membrane deformation (Figure S5d,e). To cyclic stretch PPy/PDMS membranes, a solenoid valve (V133V04, HSL Valve, Taoyuan, Taiwan) was applied to regulate vacuum pressure, and the frequency was controlled by a timer controller (ATDV‐N, ANLY, New Taipei City, Taiwan). Two electrodes were connected to two ends of PPy/PDMS to provide direct‐current electrical treatment. Electrodes were either perpendicular or parallel to the grooves of the channels, so the direction between stretching and electrical field may thus be perpendicular or parallel to each other, respectively (Figure 1c).

For the cyclic stretching, grooves of channels in depth of 0.5, 1, and 1.5 mm were applied to generate different tensile strains. A pulsatile vacuum in the frequency of 1 Hz was applied to stretch PPy/PDMS, by which C2C12 cells were cyclically stretched. These cells were stimulated 6 h a day for 4 days. Regarding the electrical stimulation, DC was applied to create an electrical field of 0.1, 0.33, or 1 V/cm to treat C2C12 cells 4 h a day for 4 days.

### 5.5 Characteristics of the bioreactor

The deformation of PPy/PDMS under vacuum was evaluated by a microcontact printing technique.^47^ Briefly, soft lithography prepared a PDMS stamp that owned an array of pillars at a distance of 160 μm. Rhodamine B was dissolved in 2 wt% of BSA to a concentration of 1 mg/mL and added 1 drop of it to the PDMS stamp. After contact with the stamp to the surface of PPy/PDMS, a fluorescent microscope (Eclipse Ti‐U, Nikon, Tokyo, Japan) was used to capture the images of stamped spots before and after stretching.

The electrical resistance of PPy/PDMS was measured by a digital multimeter (FLUKE True 115 RMS, Singapore). The distance between two multimeter probes was fixed to 1 cm and nine different points were measured per well. To evaluate the resistance of stretched PPy/PDMS membrane, the channel layer was continuously vacuumed during measurement. Furthermore, we also cyclic stretched PPy/PDMS at 1 Hz for 24 h to constantly monitor the resistance changes, so its reliability may thus be accessed.

To determine the effect of joule heating, a 1 V/cm electrical field was applied to PPy/PDMS for 4 h, and a thermal imager (DL‐770B, Dali Tech, Hangzhou, China) was used to determine its temperature distribution.

### 5.6 Culture of C2C12 myoblasts on the bioreactor and the evaluation of cell viability

In this study, C2C12 myoblasts were utilized to assess the potential of the bioreactor for muscle tissue engineering. These C2C12 cells are derived from the C2 cell line established from the thigh muscle of adult female C3H mice.^48^, ^49^ We cultured C2C12 cells in DMEM supplemented with 10 vol.% FBS. To determine the viability of cells on the bioreactor, an MTT evaluation was applied after stimulations. Medium containing 0.5 mg/mL of MTT was used to treat C2C12 cells at 37°C for 3 h. After removing the supernatant, the formazan crystals were dissolved in 1 mL of DMSO, and the absorbance was measured spectrometrically at 550 nm wavelength (Synergy H1 Hybrid reader, Biotek, Winooski, VT, USA). The readings of each group were normalized to the untreated group to determine the relative bioactivity percentage.

### 5.7 The evaluation of cell morphology

Phalloidin‐FITC staining was applied to demonstrate cell morphology, and nuclei were stained with DAPI.^24^ Fluorescent microscopy (Eclipse Ti‐U) was used to capture the images. The Image J software was utilized to assess the angle α between the stretching/electrical field direction and the long axis of the cell so that we may determine skewness according to the following definition^50^:

(1) $$\text{Skewness}=\frac{\text{number of cell with}\;\alpha >45^{{}^{\circ}}}{\text{number of cell with}\;\alpha <45^{{}^{\circ}}}.$$

### 5.8 The induction of myogenic differentiation

For myogenic differentiation induction, C2C12 cells were seeded onto the bioreactor at a density of 11,000 cells/cm^2^ for 3 days to reach a confluency of more than 80%. Then the myogenic medium was substituted to stimulate differentiation. The myogenic medium was DMEM containing 2 vol.% horse serum, 2.5 mM LiCl, and 50 nM insulin. The utilization of cyclic stretching or electrical stimulation began on the same day that the myogenic medium was substituted, and the myogenic medium was refreshed every other day.

MHC staining was used to label myotubes. Monoclonal mouse antimyosin (skeletal, fast) antibody (Cat. MFCD00145920, Sigma Aldrich) and fluorescein‐labeled goat antimouse IgG antibody (Cat. 5230–0427, Seracare, Milford, MA, USA) were sequentially applied for immunostaining and nuclei were stained with 10 μM DAPI in PBS for 10 min. The MHC‐stained area was evaluated by image software (NIS Element Basic Research, Nikon). The proportion of nuclei located within the myotubes was designated as the differentiation index.

### 5.9 Quantitative polymerase chain reaction

The TRIzol reagent (Bionovas, Toronto, Ontario, Canada) was employed to isolate the entire cellular RNA following the manufacturer's protocol, and 2 μg of RNA was converted into cDNA using the HiScript 1™ First Stand cDNA Synthesis Kit (Bionovas).

The transcribed cDNA was amplified by Real Q Plus 2x Master Mix Green with high ROX (Ampliqon, Odense M, Denmark) and monitored using a Step One Plus Real‐Time PCR System (Thermo Fisher). The primer pairs for qPCR analysis are listed in Table S1. The expression levels of the target genes were assessed by quantifying their cDNA levels, which were normalized using the levels of reference gene (GAPDH) in each reaction. In brief, the difference in cycle number between the target gene and GAPDH at the threshold fluorescence level was defined as ΔCt, which was used to normalize the transcription levels (2^−ΔCt^). These results were expressed as relative levels by comparing them to the levels of cells obtained from the bioreactor before changing to the myogenic medium at Day 0.

### 5.10 Western blot

The RIPA buffer containing protease inhibitors was applied to lyse C2C12 cells. Aliquots of lysate (50 μg) were run on a 10% SDS‐PAGE[AUTHOR: Please define (FITC, HRP, PVDF, PBST, IgG, PBS, SDS‐PAGE) in the first occurrence if necessary.] and then blotted onto a PVDF membrane. After blocking by PBS containing 0.6% Tween 20 and 5% milk, the membrane was hybridized by a monoclonal mouse antimyosin (skeletal, fast) antibody (Cat. MFCD00145920, Sigma Aldrich) overnight at 4°C. Afterward, goat anti‐mouse IgG antibody conjugated HRP (Cat. GTX213111‐01, GeneTex, Irvine, CA, USA) were applied to membrane at room temperature for 1 h. Finally, an enhanced chemiluminescence kit (ECL, R‐03031‐D25, Advansta, San Jose, CA, USA) was used to detect the HRP signal on the PVDF membrane, and the exposure was observed on x‐ray film. Through wash by PBST was applied between steps. The band intensity was analyzed by Image J software.

### 5.11 Statistical analysis

The experiments were conducted in triplicate, and the two‐tailed Student's *t*‐test was applied for statistical analysis. The errors were presented as standard deviations.

## AUTHOR CONTRIBUTIONS

**Wei‐Wen Hu:** Conceptualization (equal); funding acquisition (equal); investigation (equal); methodology (equal); resources (lead); visualization (lead); writing—original draft (lead). **Yen‐Chi Chen:** Investigation (lead); validation (lead); visualization (supporting); writing—original draft (supporting). **Chia‐Wen Tsao:** Conceptualization (equal); methodology (equal); resources (equal). **Shen‐Liang Chen:** Investigation (supporting); methodology (equal); resources (equal); validation (supporting). **Chung‐Yuh Tzeng:** Funding acquisition (equal); investigation (supporting); methodology (equal); project administration (lead); supervision (lead); writing—review and editing (lead).

## FUNDING INFORMATION

This work was supported by the Ministry of Science and Technology of Taiwan (MOST111‐2221‐E‐008‐006‐) as well as the Veterans General Hospitals and University System of Taiwan Joint Research Program (VGHUST110‐G4‐1‐2).

## CONFLICT OF INTEREST STATEMENT

The authors have no conflicts of interest to declare.

## Supporting information


**Data S1:** Supporting information.

## Data Availability

The data that support the findings of this study are available from the corresponding author upon reasonable request.
