# Supplementary material for: Development of a multifunctional bioreactor to evaluate the promotion effects of cyclic stretching and electrical stimulation on muscle differentiation
Source: Bioeng Transl Med. 2023 Dec 7;9(2):e10633. doi: 10.1002/btm2.10633 (PMC10905532; doi:10.1002/btm2.10633)
Supplement: Supplementary file 1 — Data S1: Supporting information. [file BTM2-9-e10633-s001.docx]

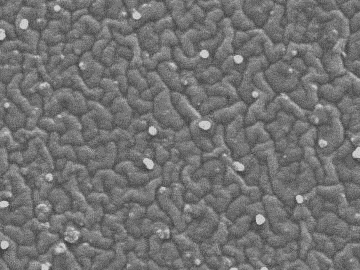
(a) (c)


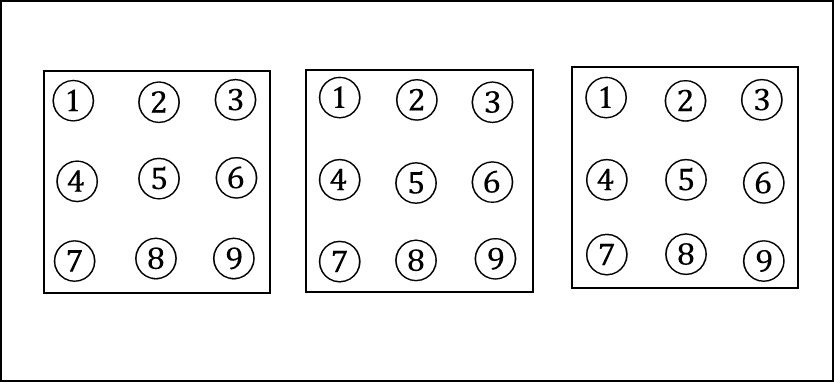


1 µm

(b)


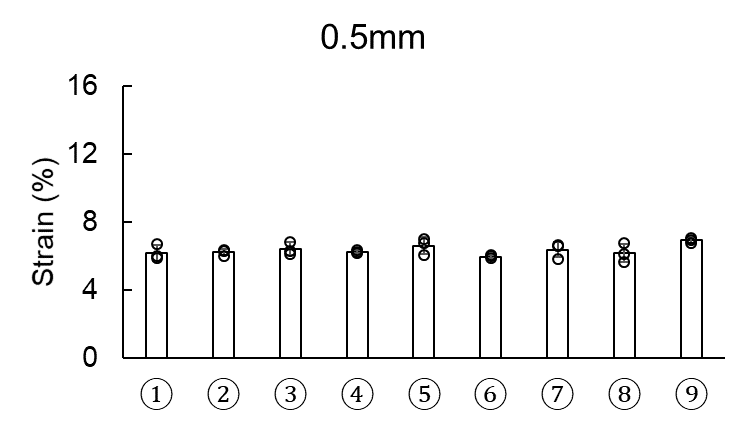

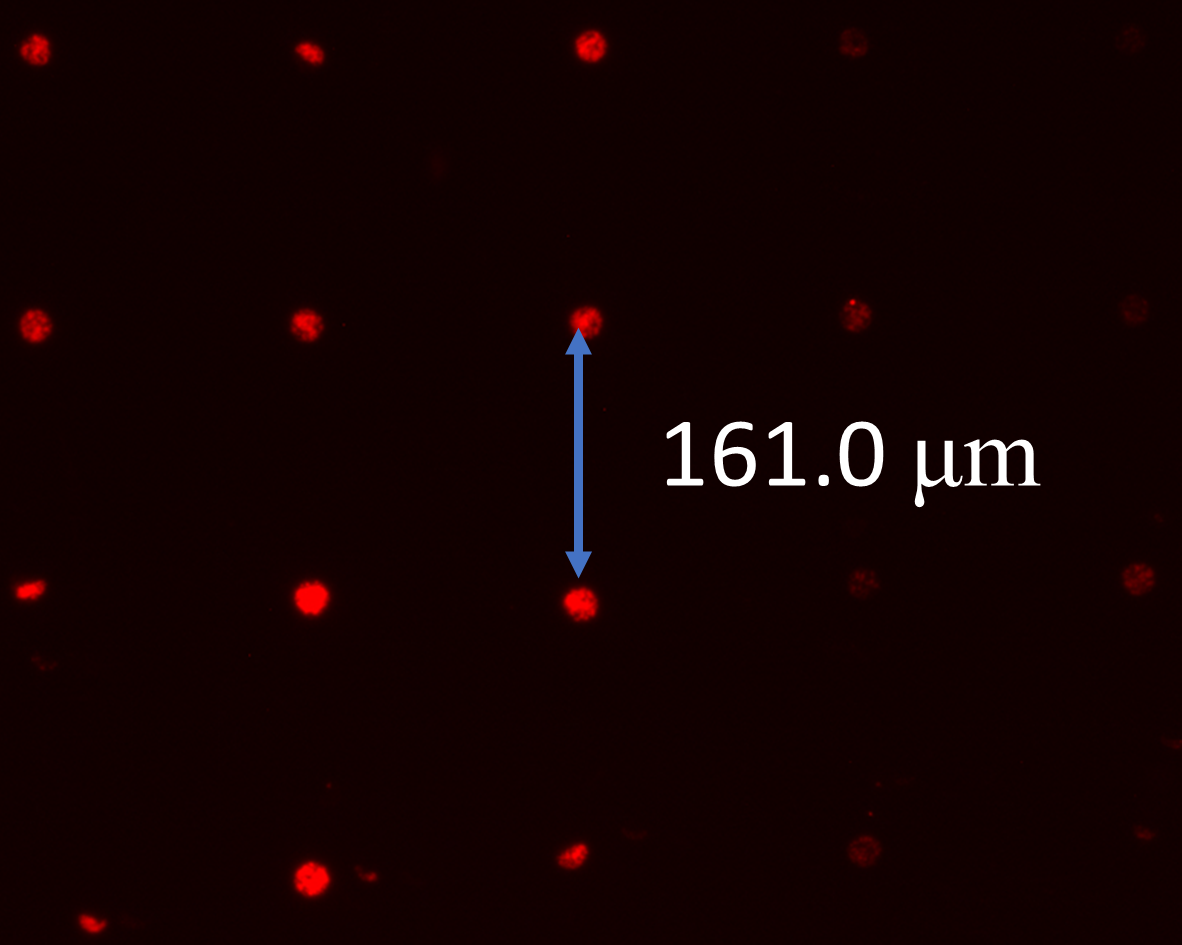

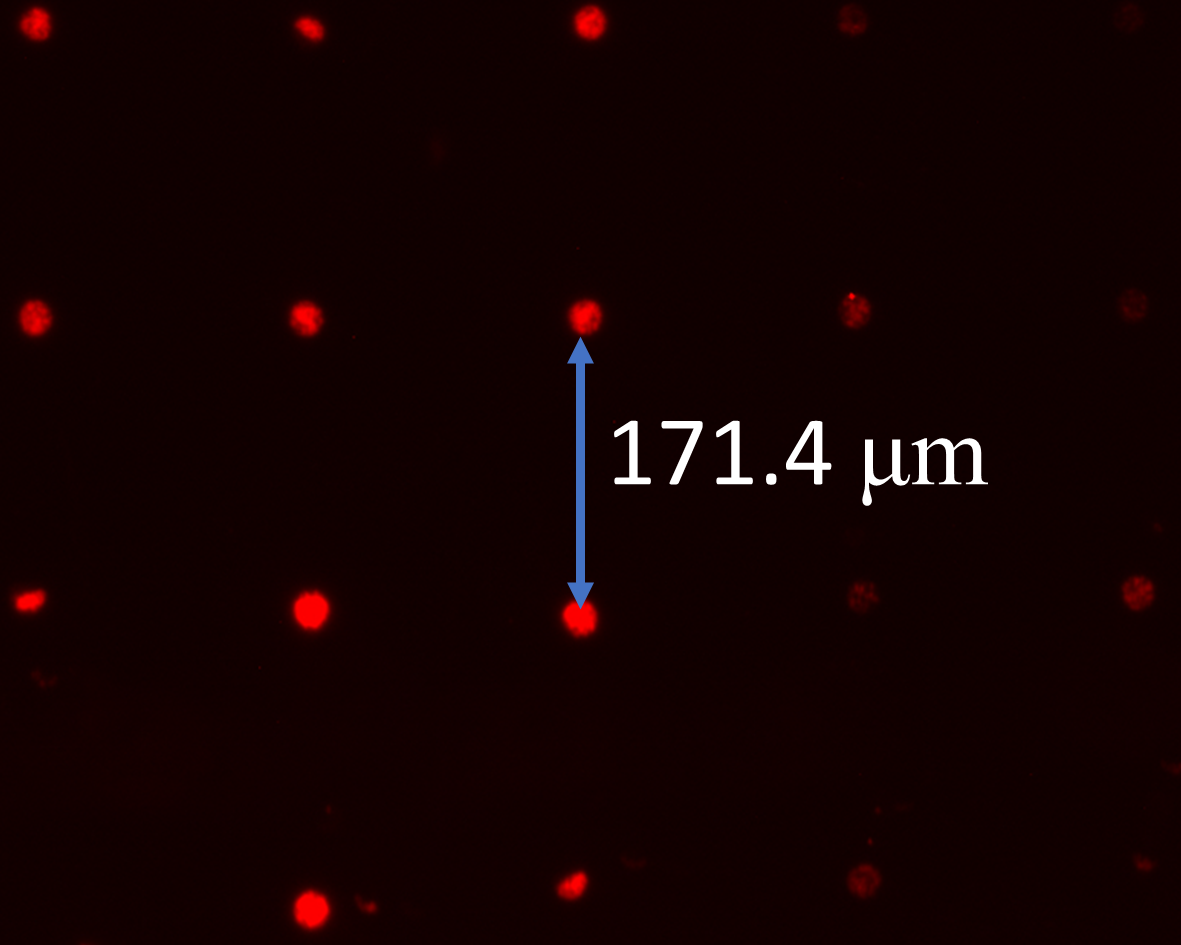


Relax Stretched


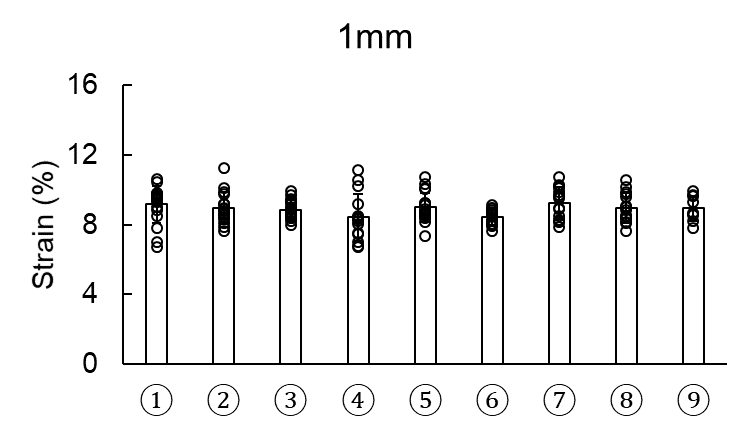

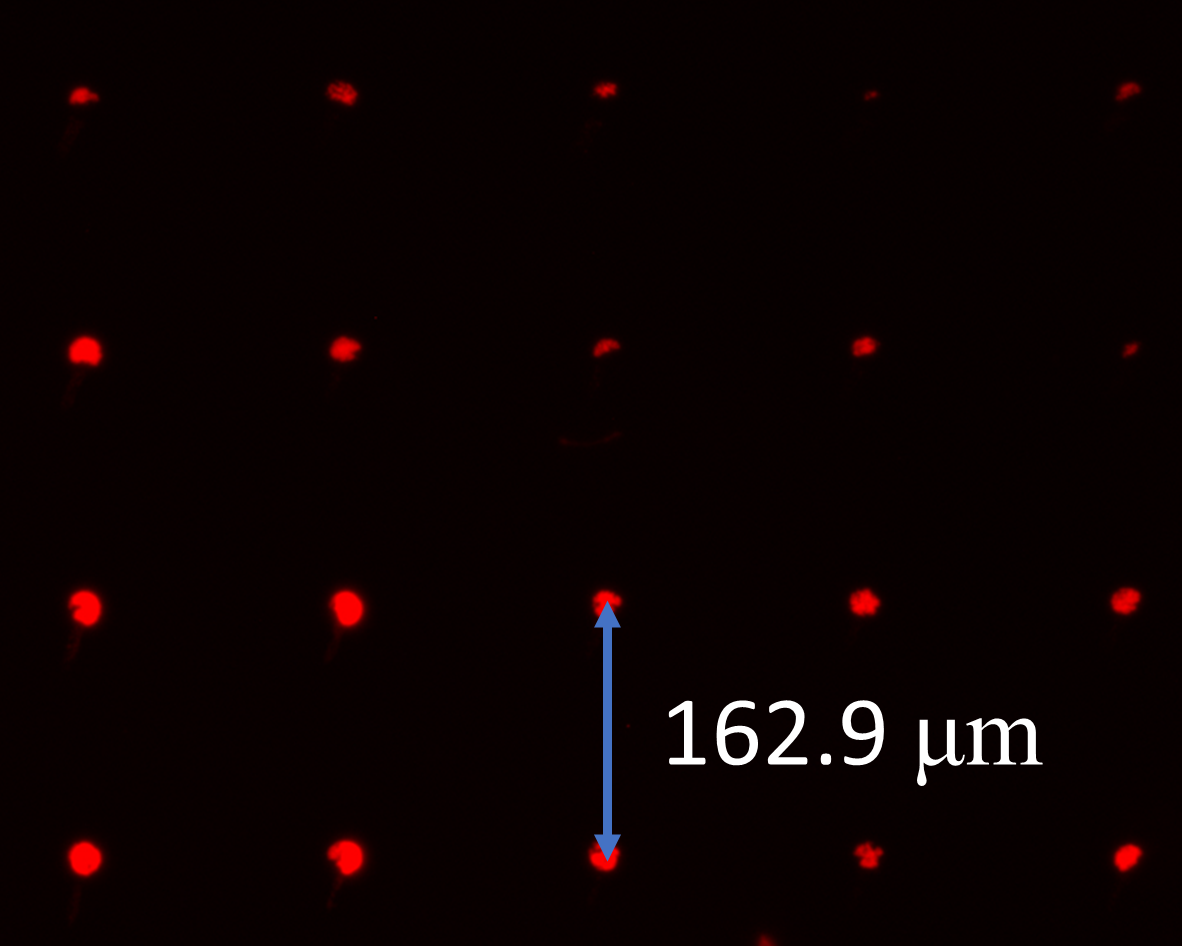

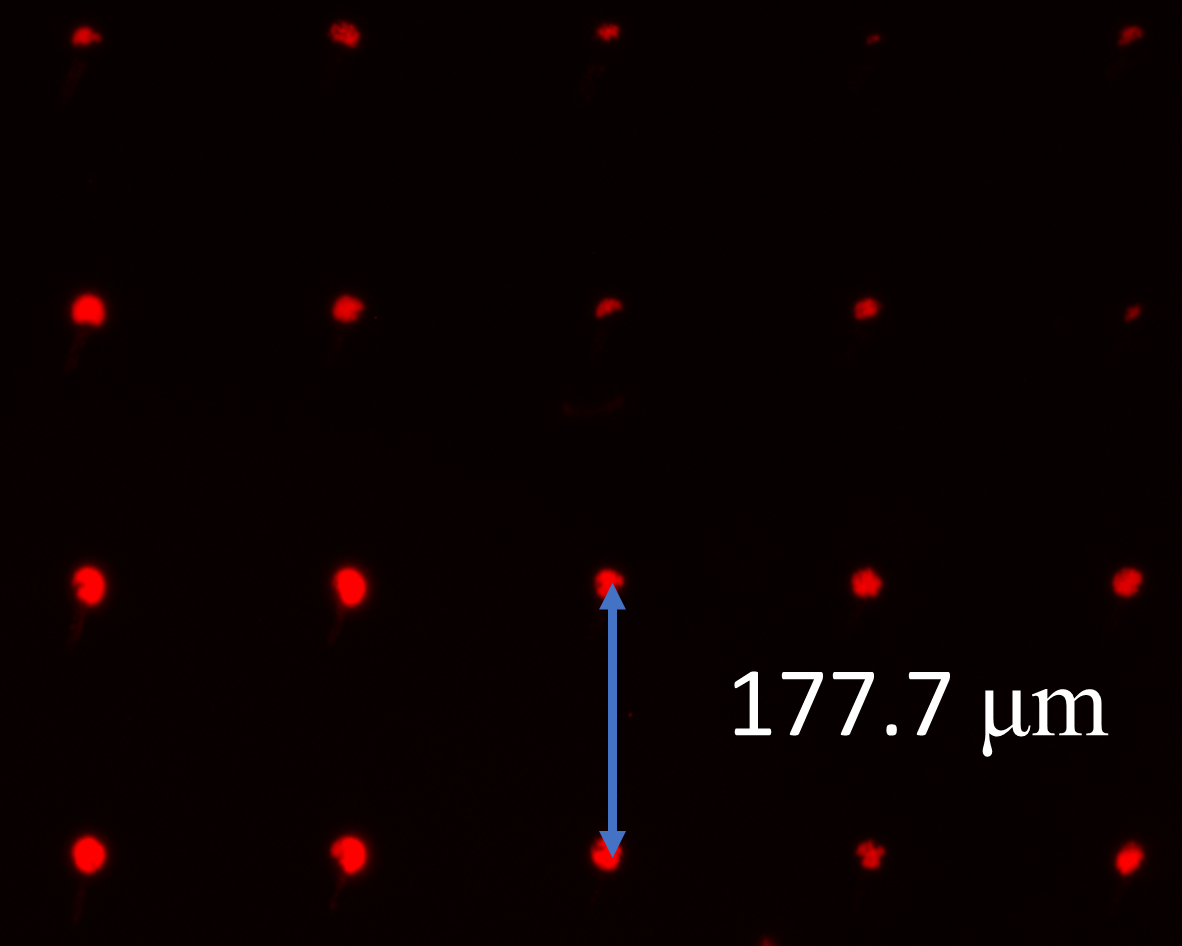


Relax Stretched


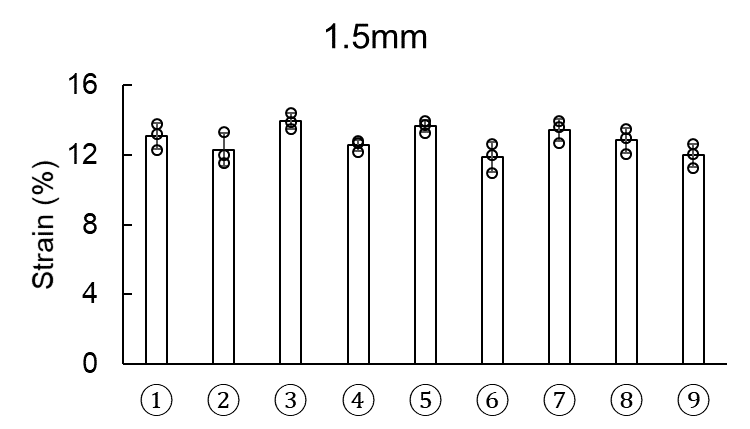

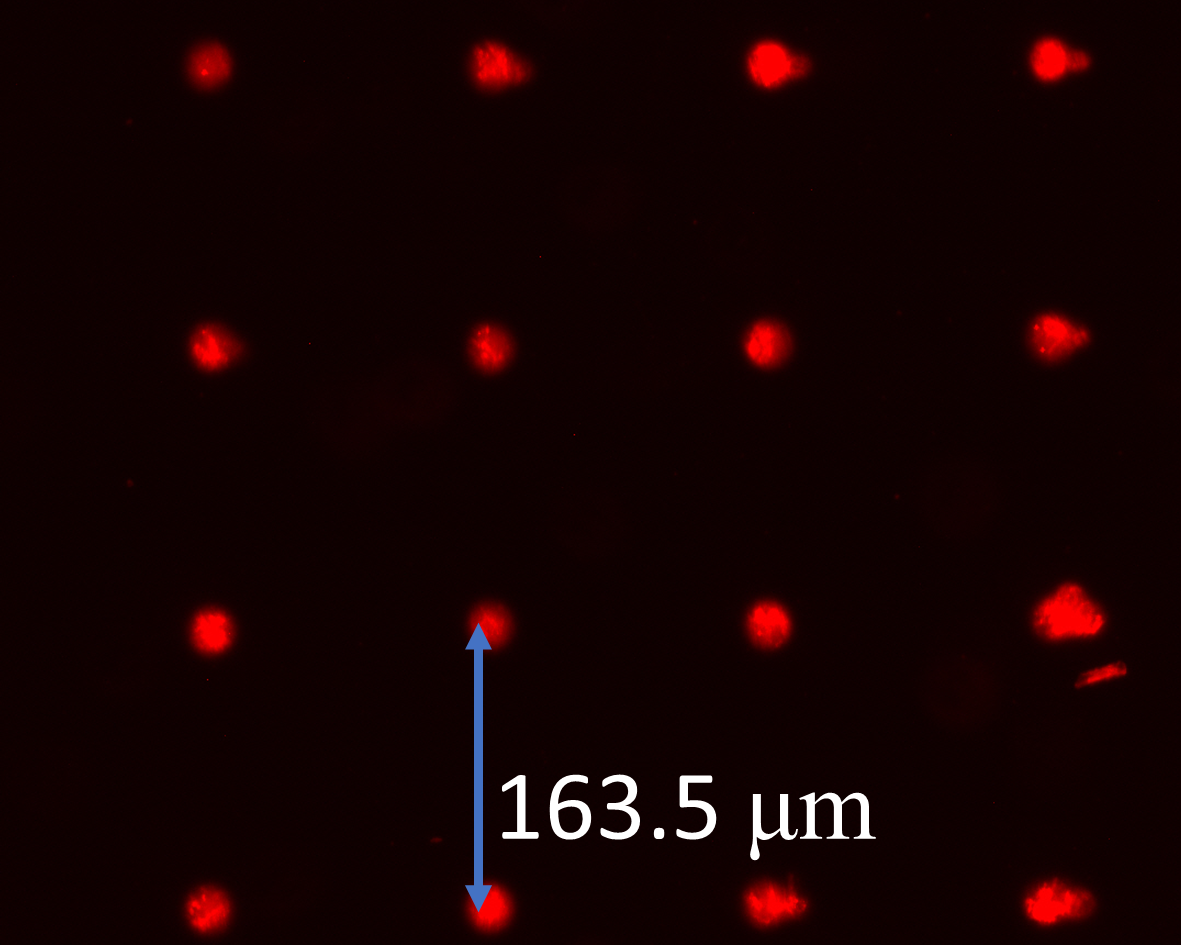

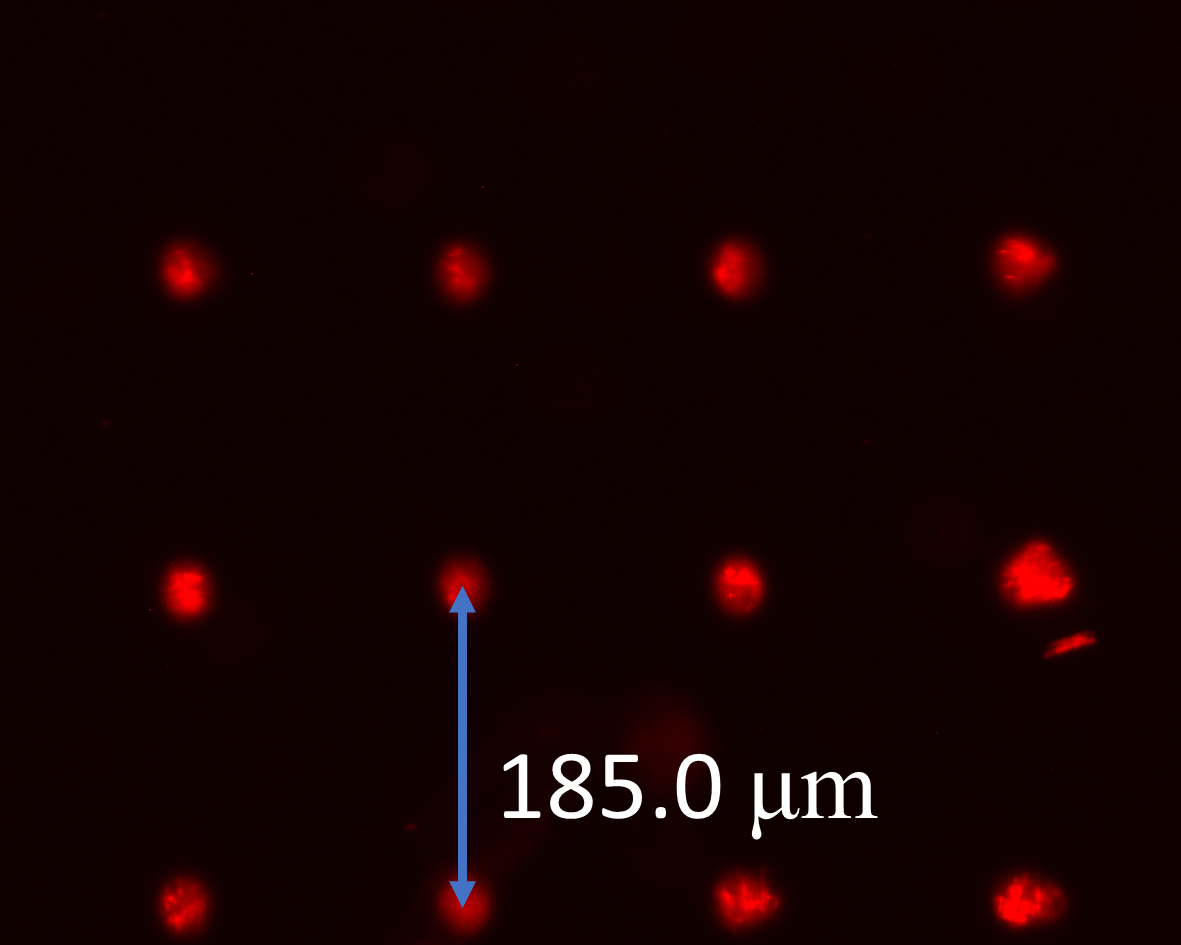


Relax Stretched

**Figure S1** *The evaluation of membrane deformation using microcontact printing assay*
(a) Nine different regions on the PPy/PDMS membrane in each well were contacted with an array of pillars with fluorescent ink. (b) The distance between spots on PPy/PDMS membrane with and without stretching were measured to determine the tensile strains generated under vacuum in bioreactors which fabricated with channel layers of different groove depths. (c) The surface morphology of PPy/PDMS after cyclic stretching for 4 h was examined by SEM (the depth of the channel was 1.5 mm, and the scale bar = 1 µm).


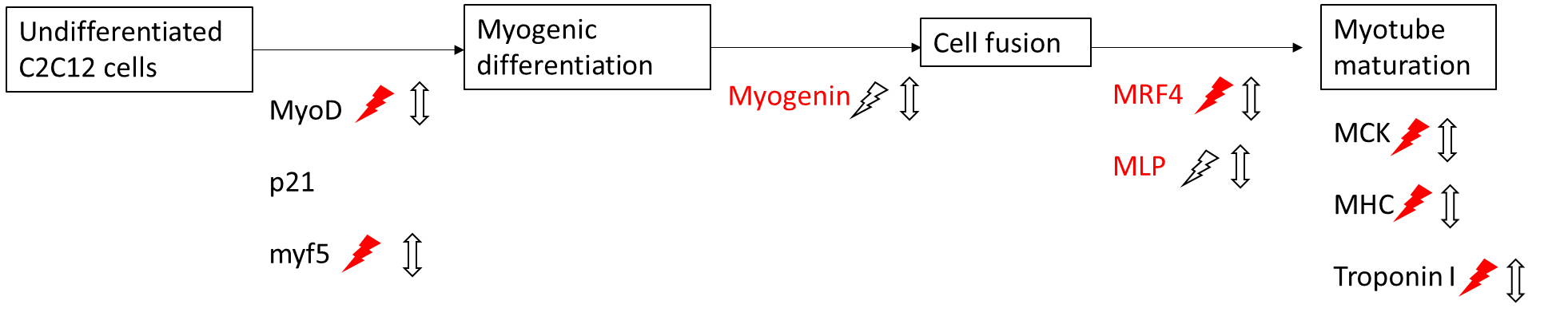


**Figure S2** *The scheme of promotion effects of stimulations on gene regulation during myogenic differentiation*
The scheme illustrates the regulation of myogenic differentiation-related genes by electrical stimulation and cyclic stretching. The lightning bolt and double arrow symbols mean that the gene can be upregulated by electrical stimulation and cyclic stretching, respectively. The red-filled lightning bolt means that gene the upregulation level of the electrical stimulation is higher than cyclic stretching. Genes marked in red exhibit synergistic effects under dual stimulation.


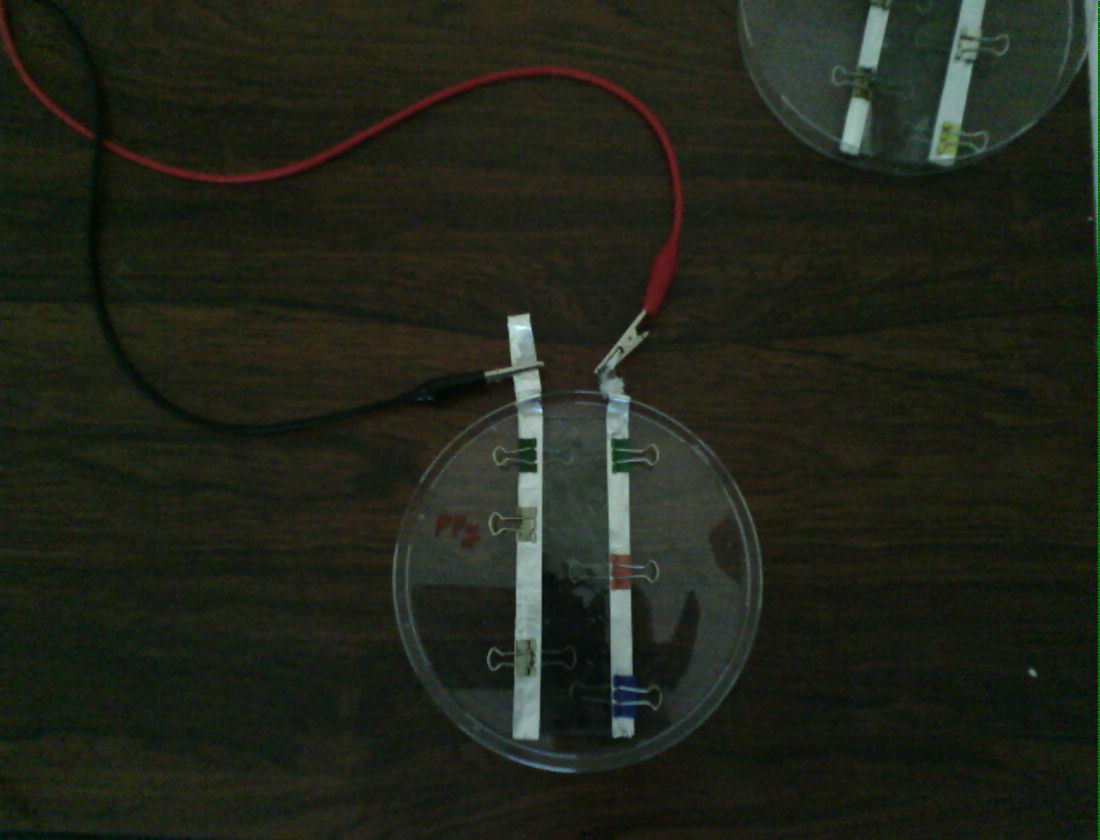

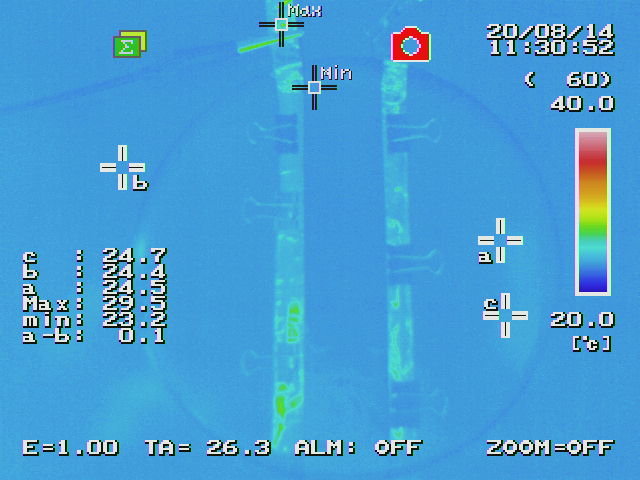


**Figure S3** *The temperature of PPy/PDMS under the electrical field of 1 V/cm*
Electrical field of 1 V/cm was applied to PPy/PDMS for 4 h (left) and a thermoal imager analyzed the temperature distribution of the PPy/PDMS. The thermal image (right) showed that the environment's temperature was 24~25 °C. The alumin foil exhibited the highest temperature (29.5 °C), but the temperature of the PPy/PDMS was almost the same as that of the environment, suggesting that our device did not elicit joule heating.


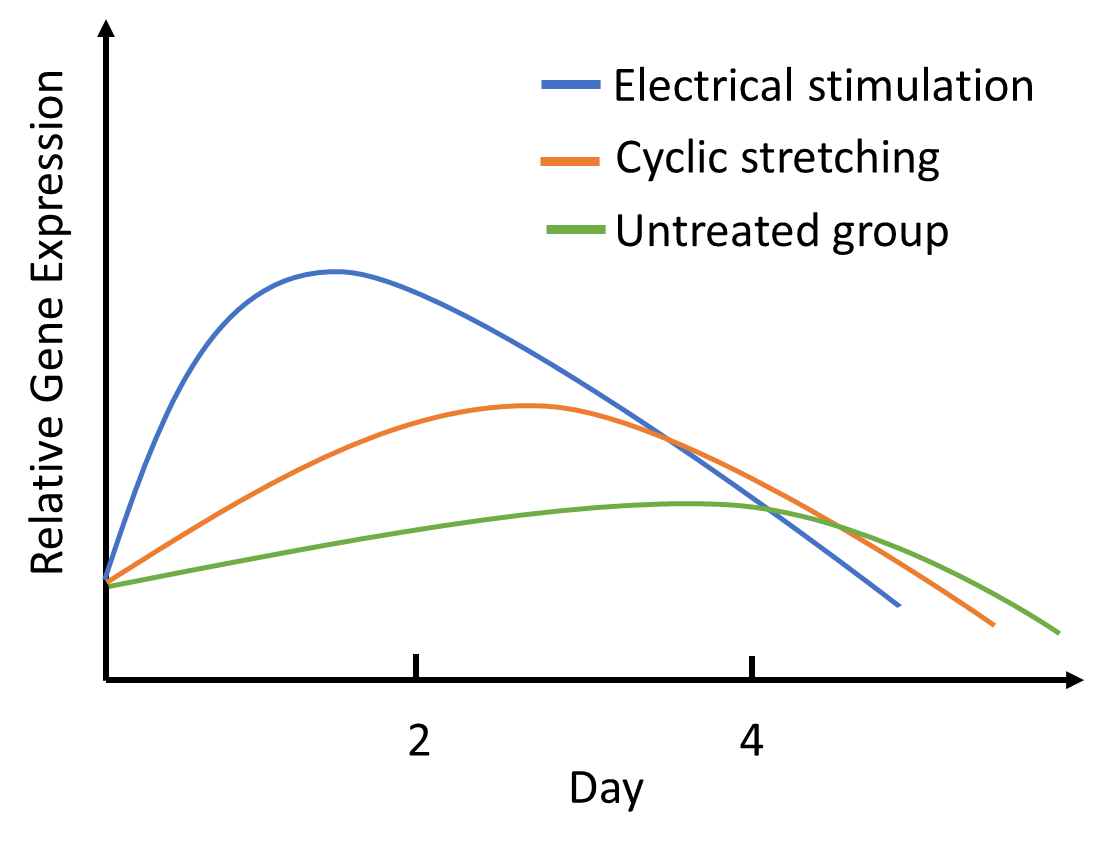


**Figure S4** *Possible effects of physical cues on the gene levels related to myogenesis*In contrast to the untreated control group, electrical stimulation and cyclic stretching both improved differentiation gene expression. Furthermore, electrical stimulation likely prompted cells to enter differentiation faster and more robust, so the amplitude and time of down-regulation are larger and earlier.

(a)

(b)

(c) (d) (e)


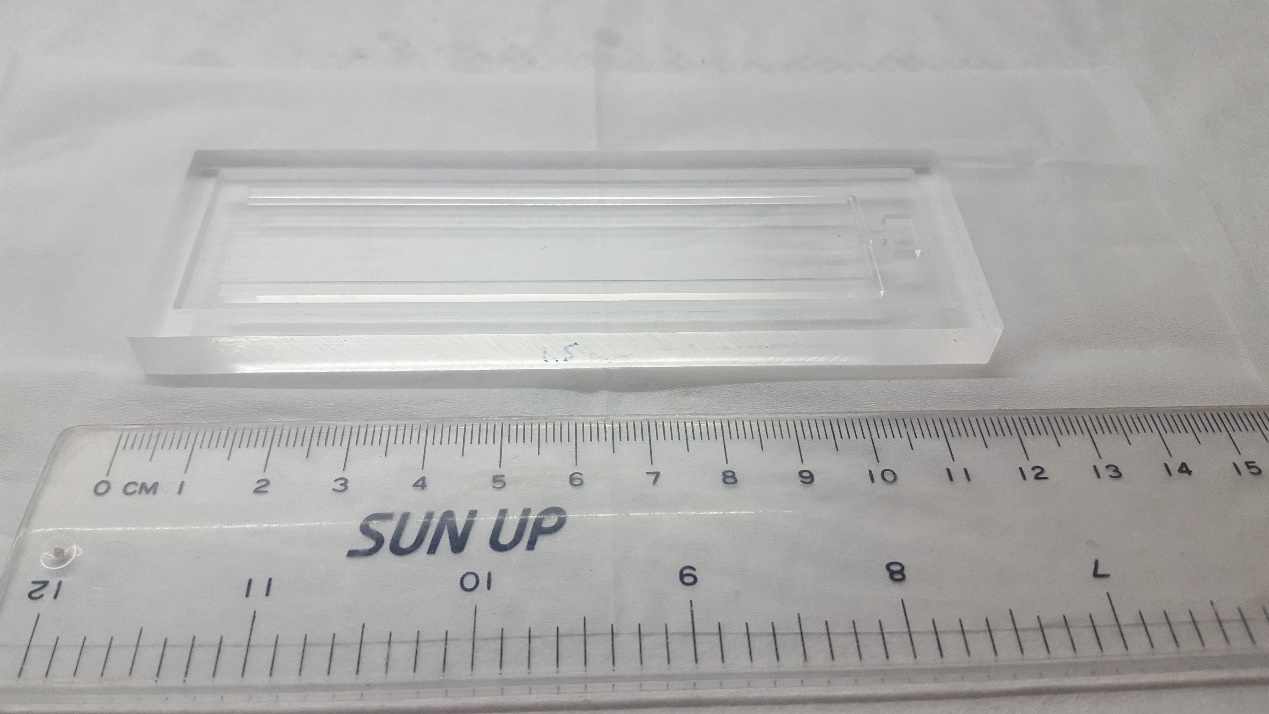

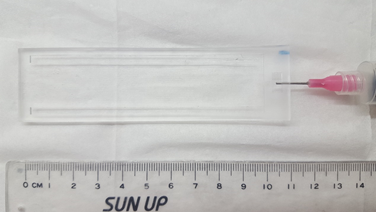

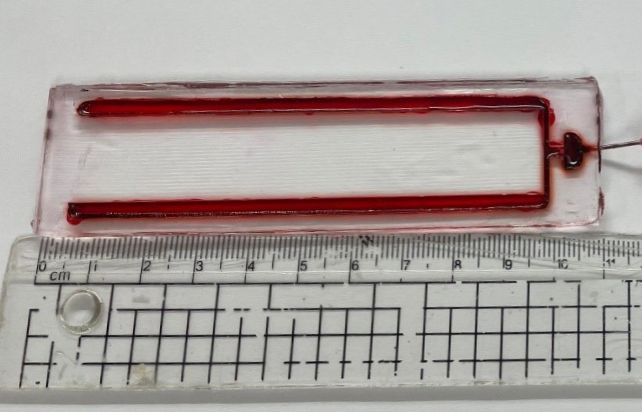


**Figure S5** *The channel layer fabrication*(a) The design of the PMMA mold. (unit = mm) (b) The channel layer was fabricated by PDMS which was cast from the mold of PMMA using CNC lathe. (c) The photo of PMMA mold. (d) The casted PDMS channel layer. A pin was applied to pouch a hole for tube insertion. (e) The channel was injected by red ink to illustrate its distribution.


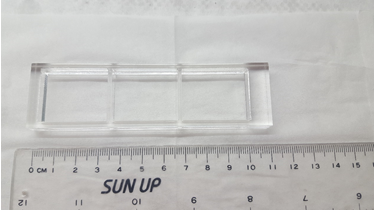


**Figure S6** *The culture-well layer fabrication*The culture-well layer was fabricated by PMMA using a CNC lathe. (a) The design of the culture well-layer. (unit = mm) (b) The photo of the culture-well layer.

**Table S1:** *The list of primers used to amplify cDNA in RT-PCR*

| **Genes** | **Primer sequences** |
| --- | --- |
| GAPDH | Forward: 5'- GGCAAATTCAACGGCACA -3'  Reverse: 5'- CCTCACCCCATTTGATGTTAGTG -3' |
| MyoD | Forward: 5'- CGGCTACCCAAGGTGGAGAT -3'  Reverse: 5'- ACCTTCGATGTAGCGGATGG -3' |
| Myf5 | Forward: 5'- TGACGGCATGCCTGAATGTA -3'  Reverse: 5'- GCTCGGATGGCTCTGTAGAC -3' |
| p21 | Forward: 5'- TCACTCTGTGTGTCTTAATTA -3'  Reverse: 5'- AGGACTGTTCCTCCGGTATAGG -3' |
| Myogenin | Forward: 5'- CTGACCCTACAGACGCCCAC -3'  Reverse: 5'- TGTCCACGATGGACGTAAGG -3' |
| MRF4 | Forward: 5'- CGAAAGGAGGAGACTAAAG -3'  Reverse: 5'- CTGTAGACGCTCAATGTAG -3' |
| MLP | Forward: 5'- TGGGTTTGGAGGGCTTAC -3'  Reverse: 5'- CACTGCTGTTGACTGATAGG -3' |
| MHC | Forward: 5'- AGCAGACGGAGAGGAGCAGGAAG-3'  Reverse: 5'- CTTCAGCTCCTCCGCCATCATG -3' |
| MCK | Forward: 5'- CAATAAGCTTCGCGATAAGGAG -3'  Reverse: 5'- GATGGGATCAAACAGGTCCTTG -3' |
| Troponin I | Forward: 5'- GCCTATGCGCACACCTTTG -3'  Reverse: 5'- CGGGTACCATAAGCCCACACT -3' |
